# XBP1 mitigates aminoglycoside-induced endoplasmic reticulum stress and neuronal cell death

**DOI:** 10.1038/cddis.2015.108

**Published:** 2015-05-14

**Authors:** N Oishi, S Duscha, H Boukari, M Meyer, J Xie, G Wei, T Schrepfer, B Roschitzki, E C Boettger, J Schacht

**Affiliations:** 1 Department of Otolaryngology, Kresge Hearing Research Institute, University of Michigan, Ann Arbor, MI, USA; 2 Institut für Medizinische Mikrobiologie, Universität Zürich, Zürich, Switzerland; 3 Functional Genomics Center Zurich, ETH Zürich, Universität Zürich, Zürich, Switzerland

## Abstract

Here we study links between aminoglycoside-induced mistranslation, protein misfolding and neuropathy. We demonstrate that aminoglycosides induce misreading in mammalian cells and assess endoplasmic reticulum (ER) stress and unfolded protein response (UPR) pathways. Genome-wide transcriptome and proteome analyses revealed upregulation of genes related to protein folding and degradation. Quantitative PCR confirmed induction of UPR markers including C/EBP homologous protein, glucose-regulated protein 94, binding immunoglobulin protein and X-box binding protein-1 (XBP1) mRNA splicing, which is crucial for UPR activation. We studied the effect of a compromised UPR on aminoglycoside ototoxicity in haploinsufficient XBP1 (XBP1^+/−^) mice. Intra-tympanic aminoglycoside treatment caused high-frequency hearing loss in XBP1^+/−^ mice but not in wild-type littermates. Densities of spiral ganglion cells and synaptic ribbons were decreased in gentamicin-treated XBP1^+/−^ mice, while sensory cells were preserved. Co-injection of the chemical chaperone tauroursodeoxycholic acid attenuated hearing loss. These results suggest that aminoglycoside-induced ER stress and cell death in spiral ganglion neurons is mitigated by XBP1, masking aminoglycoside neurotoxicity at the organismal level.

Translational fidelity is maintained throughout all three domains of life (archea, bacteria and eukaryota), suggesting a high selective pressure during evolution to minimize errors in protein synthesis.^1^ In bacteria, erroneous protein synthesis induces protein misfolding.^2^ In higher eukaryotes, protein misfolding results in endoplasmatic reticulum (ER) stress and initiates the unfolded protein response (UPR), a cascade of integrated pathways regulating gene expression. The UPR^ER^ is mediated by three ubiquitously expressed transmembrane proteins in the ER: inositol-requiring enzyme 1 (IRE1), PKR-like ER kinase (PERK) and activating transcription factor 6 (ATF6).^3, 4, 5, 6, 7^ Under normal conditions, the luminal domains of IRE1, PERK and ATF6 are bound by the ER chaperone-binding immunoglobulin protein (BiP), which inhibits self-dimerization and activation of the cytosolic domain.^8, 9^ Under ER stress, BiP is released resulting in dimerization of IRE1 and ATF6 and oligomerization of PERK, initiating the UPR signaling cascades.^8, 9^ The initial UPR response is protective, increasing the expression of chaperone proteins promoting refolding and, if unsuccessful, the degradation of misfolded proteins.^10, 11, 12, 13^ Prolonged or severe stress triggers additional pathways that eventually lead to cellular apoptosis.^14, 15, 16^

Aminoglycoside antibiotics are well known to affect translational fidelity in bacteria and lower eukaryotes^17, 18, 19, 20^ but only few reports suggest that aminoglycoside antibiotics may also induce misreading in higher eukaryotes.^21, 22, 23^ Aminoglycoside-mediated readthrough activity has been exploited for therapy of human genetic diseases associated with premature stop codons.^24, 25, 26, 27^ In addition, aminoglycosides have been shown to induce apoptosis in human cell cultures, accompanied by ER stress and mitochondrial cytochrome c release.^28, 29^ It was suggested that the observed ER stress could be the result of protein misfolding, reflecting aminoglycoside-induced mistranslation.^28^ Despite this potential for misreading induced by aminoglycosides in eukaryotes, aminoglycoside treatment in experimental animals and in patients is well tolerated. Side effects are highly organ specific, limited to the kidney and the inner ear,^30^ while toxicity to the nervous system is not evident even in long-term aminoglycoside administration.^31^ In the case of ototoxicity, the primary drug target are the sensory hair cells, as convincingly demonstrated in various animal models, regardless of whether the drug is given systemically^32^ or directly introduced into the cochlea.^33^ Degeneration of spiral ganglion cells (SGCs) observed after ototoxic dosages of aminoglycosides are thought to occur only as a sequel to the loss of sensory hair cells in the vast majority of cases. Surprisingly, however, a few analyses of human temporal bones have suggested that spiral ganglia can be affected by aminoglycosides without overt insult to the hair cells.^34, 35^ This rare pathology, unexplained by the treatment modus, suggests individual variability possibly based on genetic factors.

Prompted by the anecdotal reports of aminoglycoside-induced selective spiral ganglion damage and the potential of aminoglycosides to induce mistranslation, the objective of this study was to assess the contribution of ER stress to ototoxicity. We first investigated aminoglycoside-induced misreading and UPR responses in HEK293 cells *in vitro*. Next, we examined the role of ER stress in ototoxicity in cochlear organ cultures of CBA/J mice. Finally, we used an *in vivo* mouse model^36^ with a compromised ER stress response because of X-box binding protein-1 (XBP1) haploinsufficiency^37^ in order to probe potential links between aminoglycoside neurotoxicity, translation fidelity and protein misfolding.

## Results

### Aminoglycosides alter translation fidelity

**Figure 1:**
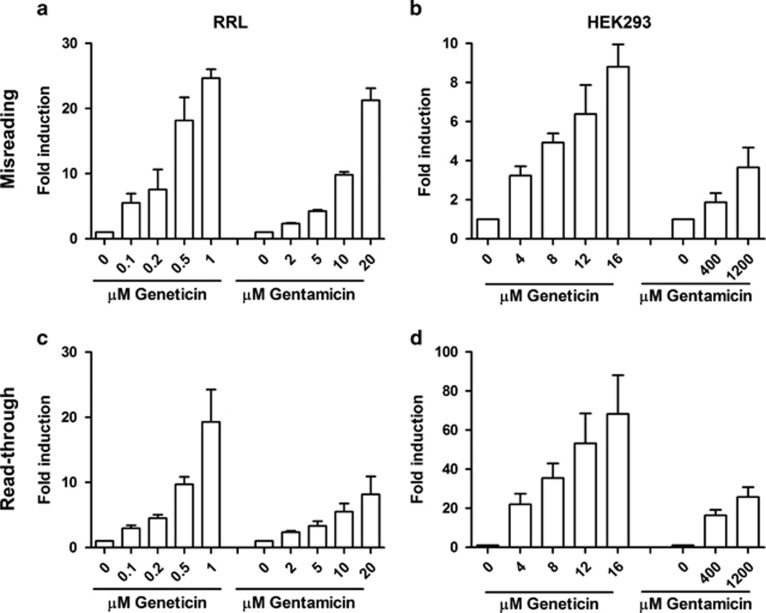
Aminoglycoside-induced mistranslation. (**a–b**) Misreading and (**c–d**) readthrough was measured in RRL (**a** and **c**) and HEK wild-type cells (**b** and **d**). Results are derived from the ratio hFluc/hRluc, given in fold induction. Untreated samples are set as 1 (*n*=3; ±S.E.M.)

Drug-induced inhibition of translation was used to assess aminoglycoside activity on the eukaryotic ribosome. IC50 values were 0.3 *μ*M for geneticin and 9.8 *μ*M for gentamicin in the cell-free translation assays with rabbit reticulocyte lysate (RRL), and 4.4 *μ*M for geneticin and 812 *μ*M for gentamicin in assays with intact HEK293 cells (Supplementary Figures S1a and b). The ability of the drugs to induce mistranslation was analyzed using sensitive gain-of-function dual-luciferase assays to assess near-cognate misreading and stop codon readthrough. Near-cognate misreading was studied using constructs with substitution of amino-acid 245 in the active site of mutated firefly luciferase (wild-type His CAC→near-cognate Arg CGC), which results in loss of enzymatic activity with enzymatic function restored by misreading; stop codon readthrough was determined using constructs with in-frame stop codons abolishing firefly luciferase activity. Both geneticin and gentamicin decreased ribosomal accuracy in cell-free translation assays (RRL) and in HEK cells in a dose-dependent manner (Figure 1). Misreading was induced up to 25-fold in RRL and up to 8.5-fold in HEK cells compared with untreated controls; readthrough was induced up to 20-fold in RRL and up to 70-fold in HEK cells compared with untreated controls (Figure 1). In HEK cells transfected with the aminoglycoside phosphotransferase APH(3′), the geneticin-induced but not the gentamicin-induced translation inhibition and mistranslation were abrogated (Supplementary Figures S1c and d), consistent with the selectivity of the enzyme to inactivate geneticin but not gentamicin.^38, 39^ Aminoglycoside-treated and -untreated HEK wild-type cells showed similar metabolic activities and viability at both the 24-h and 48-h time points (Supplementary Figures S1e and f).

### Aminoglycosides induce genome-wide upregulation of cellular folding capacity

In order to study the cellular response to aminoglycoside-induced mistranslation, we used whole-genome transcriptomic and proteomic analyses. A microarray analysis of geneticin-treated *versus* -untreated cells revealed a broad transcriptional response totaling 705 genes (selected for a fold change >1.2, Benjamini–Hochberg corrected *P*-value <0.05; Supplementary Figure S2a). Protein folding and transcription were among the most enriched functional ontologies (Supplementary Figure S2b), including the induction of the ER-specific chaperones BiP (HSPA5), glucose-regulated protein 94 (GRP94; HSP90B1), calreticulin (CALR), GRP110 (HYOU1), ERdj3 (DNAJB11) and ERdj6 (DNAJC3), the ER foldases PDIA3 (ERp57), PDIA4 (ERp70), ERp44 and FKBP7, and the N-linked glycosylation factor SDF2L1. Similarly, ER-associated degradation (ERAD) components such as VCP (p97), Derlin2 (DERL2) and Herp (HERPUD1) were significantly upregulated (Supplementary Figure S2c). This transcriptional response indicates a broad increased folding and degradation capacity in the ER. In addition, a large number of cytosolic chaperones^40^ were upregulated, such as members of the Hsp40, Hsp70, Hsp90 and Hsp110 families and to a lesser extent foldases (peptidyl-prolyl cis/trans isomerases and protein disulfide isomerases; Supplementary Figures S2d and e), indicating an increased folding capacity in the cytosol. Supplementary Table S1 lists the genes included in the analysis. The microarray data have been deposited in NCBI's Gene Expression Omnibus and are accessible through GEO Series accession number GSE57198 (http://www.ncbi.nlm.nih.gov/geo/query/acc.cgi?acc=GSE57198).

**Figure 2:**
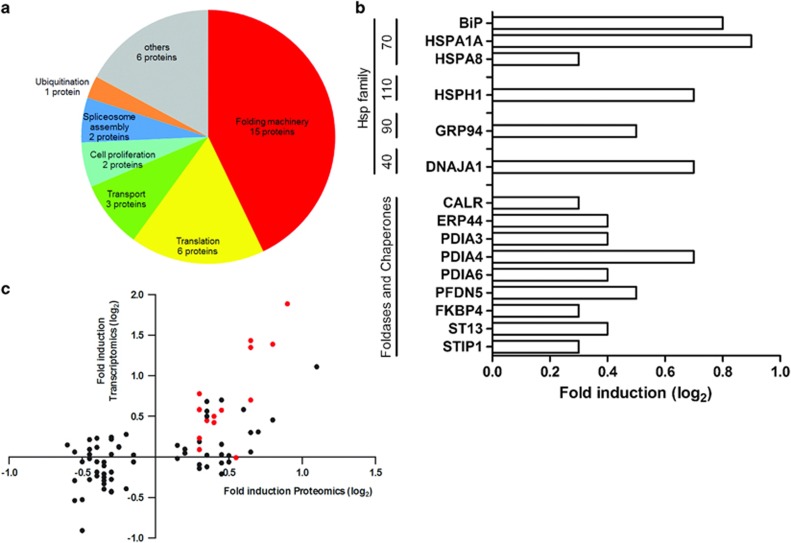
Proteomic analysis of geneticin-treated HEK wild-type cells. (**a**) Thirty-five upregulated proteins (Bonferroni-corrected *P*-value <0.05, log_2_ FC >0.3) were grouped according to their biological function. (**b**) Upregulation of the geneticin-induced heat shock proteins, chaperones and foldases (Bonferroni-corrected *P*-value <0.05, log_2_ FC >0.3). (**c**) Comparison of the significantly regulated proteins (Bonferroni-corrected *P*-value <0.05) and their corresponding mRNA fold induction. The upregulated proteins of the folding machinery are shown in red

Proteome analysis found 77 proteins to be regulated by geneticin (Bonferroni-corrected *P*-value <0.05). When applying a minimum fold induction of 0.3 (log2 scale) we identified 35 proteins that were upregulated. Grouping according to function revealed a predominance of proteins involved in protein folding (Figure 2a). Proteins associated with the ER and cytoplasmic UPR, such as BiP, GRP94, calreticulin, foldases, and members of the Hsp70, Hsp90, Hsp110 and Hsp40 families, were also upregulated (Figure 2b). Comparison with corresponding mRNA levels showed an upregulation of the folding machinery both at the transcriptomic and the proteomic level (Figure 2c). The mass spectrometry proteomics data have been deposited to the ProteomeXchange Consortium via the PRIDE partner repository with the data set identifier PXD000933 and DOI 10.6019/PXD000933.

### Aminoglycosides induce the UPR

**Figure 3:**
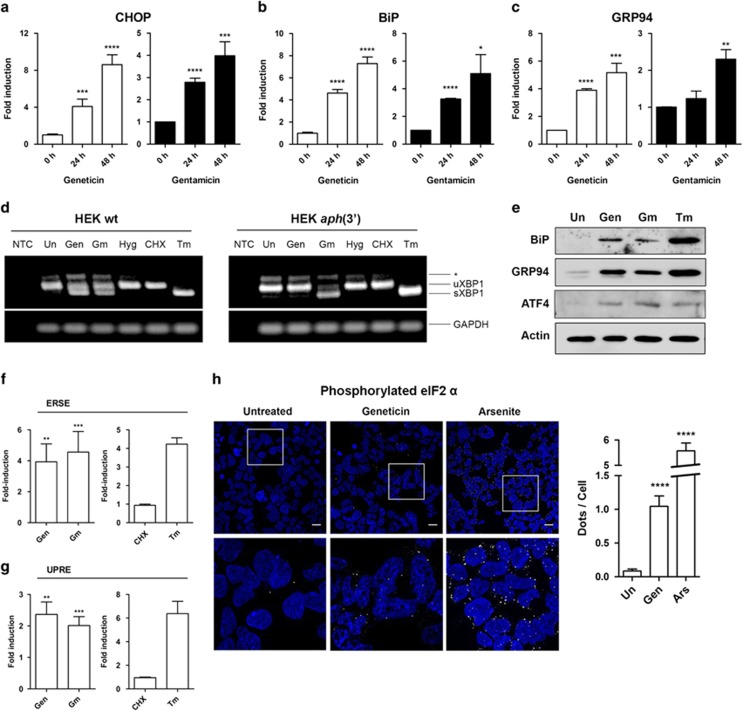
Aminoglycosides induce the UPR. (**a–c**) qPCR analysis. HEK wild-type cells were treated with geneticin (16 *μ*M) or gentamicin (400 *μ*M) and incubated for the indicated times. Expression of mRNA for CHOP (**a**), BiP (**b**) and GRP94 (**c**) is shown. Means+S.D. of fold induction are presented relative to 0 h (untreated) sample (*n*=3); *P<0.05; **P<0.01; ***P<0.005; ****P<0.001. (**d**) XBP1-splicing assay. HEK wild-type or HEK *aph*(3′) cells were treated with geneticin (16 *μ*M), gentamicin (1250 *μ*M), hygromycin (2 *μ*M), cycloheximide (2 *μ*M), tunicamycin (5 *μ*g/ml) for 24 h or left untreated. NTC, no template control. PCR products of XBP1 were analyzed by gel electrophoresis; unspliced and spliced versions of XBP1 are indicated. Tunicamycin was a positive control to induce ER stress; GAPDH was a loading control. The asterisk indicates the position of a hybrid amplicon.^15^ (**e**) Western blot analysis. HEK wild-type cells were treated with geneticin (16 *μ*M) or gentamicin (400 *μ*M) and incubated for 24 h. Ten micrograms of total protein were loaded and BiP, GRP94 and ATF4 were detected by immunoblotting using specific antibodies. *β*-Actin was used as a loading control and tunicamycin (2.5 μg/ml) as a positive control. (**f** and **g**) Reporter assays. HEK cells were transfected with luciferase reporter plasmids (**f**) UPRE (reporter for ATF6 activity) or (**g**) ERSE (reporter for ATF6 and XBP1 activity). Cells were treated with geneticin (16 μM) or gentamicin (800 *μ*M) for 24 h. Cycloheximide (16 *μ*M) was used as a negative control, and tunicamycin (2.5 *μ*g/ml) as a positive control for eliciting UPR. Luciferase activities were determined and the Fluc/Rluc ratios were calculated. Untreated samples are set as 1 and fold inductions are given (*n*=3–6, ±S.E.M.). **P<0.01, ***P<0.005. (**h**) Phosphorylated eIF2*α* was detected by immunofluorescence. HEK wild-type cells were treated with geneticin (16 *μ*M) for 24 h or arsenite (0.5 mM) for 1 h as a positive control. Scale bars: 40 *μ*m. The lower panels show insets in higher magnification. Bar graph indicates quantification of p-eIF2*α* immunofluorescence (*n* number of cells; *n*_Un_=540; *n*_Gen_=249; *n*_Ars_=648); ****P<0.001. Ars, arsenite; CHX, cycloheximide; Gen, geneticin; Gm, gentamicin; Hyg, hygromycin; Tm, tunicamycin; Un, untreated

To corroborate the results of the microarray analysis, mRNA levels of selected UPR genes were further analyzed by quantitative PCR and corresponding protein levels assessed by western blotting. Geneticin and gentamicin-induced mRNA expression of C/EBP homologous protein (CHOP), GRP94 and BiP in a time-dependent manner (Figures 3a–c). Increased protein levels of the two ER chaperones BiP and GRP94, as well as the transcription factor ATF4, which is regulated at the translational level,^41^ were observed in geneticin- and gentamicin-treated cells by western blotting (Figure 3e). As a further element of the UPR, we studied splicing of XBP1 mRNA, which is central for UPR activation.^11^ Both geneticin and gentamicin induced XBP1 splicing (Figure 3d). In contrast, XBP1 splicing was induced neither by the non-misreading aminoglycoside hygromycin^42^ nor by cycloheximide, an inhibitor of ribosomal translocation,^43^ indicating that XBP1 splicing depends on misreading and not on inhibition of translation. Furthermore, the presence of APH(3′) in HEK cells abrogated geneticin-induced but not gentamicin-induced XBP1 splicing.

The activity of transcription factors XBP1 and ATF6 was examined using reporter plasmids UPRE (p5xATF6-GL3-luc) and ERSE (pGL3-GRP78P(-132)-luc).^44, 45^ The UPRE reporter is specific for ATF6 activity, the ERSE reporter is regulated by both ATF6 and XBP1.^44, 45^ Both reporters showed a robust induction by geneticin and gentamicin (Figures 3f and g). Cycloheximide failed to induce any reporter activity consistent with the XBP1-splicing results (Figures 3d, f and g). The PERK signaling branch was investigated by assessing the formation of stress granules, cytosolic protein aggregates composed of 48 S preinitiation complexes and other factors. Stress granules are induced upon activation of PERK and phosphorylation of eIF2*α*.^46^ Treatment of HEK wild-type cells with geneticin for 24 h increased immunostaining against phosphorylated eukaryotic initiation factor 2 alpha (p-eIF2*α*) in a dotted cytosolic distribution consistent with the formation of stress granules (Figure 3h). Arsenite treatment served as a positive control. A similar robust induction of UPR by aminoglycosides was observed in HeLa cells (Supplementary Figure S3).

### Gentamicin induces ER stress in SGCs but not in auditory hair cells

**Figure 4:**
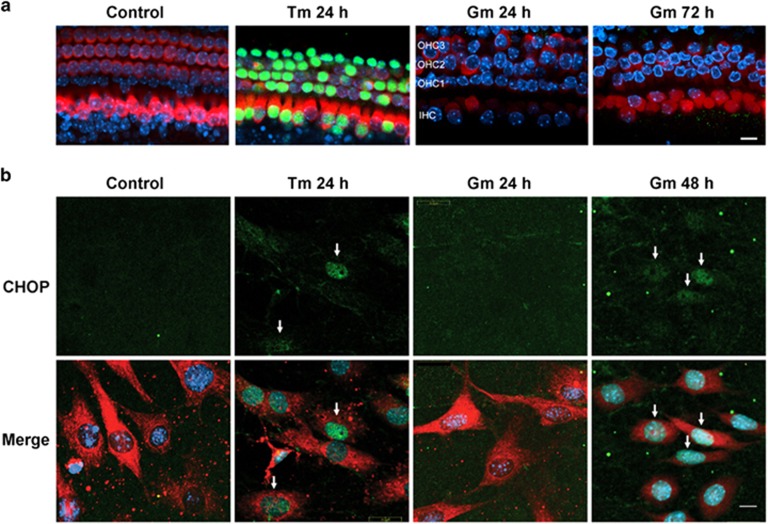
ER stress in cochlear tissues. (**a**) Tunicamycin but not gentamicin causes ER stress in hair cells. Tunicamycin (0.07 *μ*g/ml) induced the specific ER stress-associated pro-apoptotic factor, CHOP (green), in the nuclei of hair cells in organ of Corti explants by 24 h. In contrast, CHOP was not observed in any part of the organ of Corti throughout the entire time course of gentamicin treatment (3.5 *μ*M) until hair cell death. Segments shown are from the basal turn. Green: CHOP (GADD 153 antibody), red: myosin 7a antibody, blue: Hoechst 33342 staining for nuclei. The focal plane is at the nuclear level of outer hair cells leaving some regions stained against myo7a out of focus. The figure represents three different explants at each time point. Scale bar (Gm): 10 *μ*m. (**b**) Gentamicin induces ER stress in SGCs. Tunicamycin (0.07 *μ*g/ml) treatment for 24 h induced CHOP in the nuclei of SGCs (arrows). With gentamicin treatment (3.5 *μ*M), CHOP appeared in the nuclei of SGCs by 48 h (arrows). Green: CHOP (GADD 153 antibody), red: neuronal class III *β*-tubulin staining for SGCs, blue: Hoechst 33342 staining for nuclei. The figure represents three different explants at each time point. Scale bar, 10 *μ*m

To study the response of auditory hair cells to ER stress, we first used tunicamycin, an established ER stress inducer in early post-natal cochlear explants of the CBA/J mouse, a common strain for auditory research. Preliminary experiments (data not shown) with hair cell counts on surface preparations had established incubation with 0.07 *μ*g/ml tunicamycin as a suitable treatment with hair cell death beginning at 48 h and encompassing 50% of cells by 72 h. The ER stress-associated pro-apoptotic factor CHOP already appeared after 8 h of incubation with tunicamycin and was expressed in the nuclei of most hair cells by 24 h (Figure 4a and Supplementary Figure S4). Staining mostly had disappeared at 48 h (Supplementary Figure S4) when loss of hair cells became apparent, implicating CHOP as an indicator of impending hair cell death. In the same explant model, treatment with gentamicin produced significant loss of hair cells with the pattern of loss showing the typical progression of aminoglycoside damage^47^ causing most destruction in the base (Supplementary Figure S5), whereas inner hair cells (IHCs) were mostly spared. Despite continuing and increasing cell death, CHOP was not observed throughout the entire time course up to 72 h (Figure 4a).

The response of ganglion neurons to ER stress was studied in SGCs that were harvested from the base to the middle of the modiolus of cochlear explants and similarly treated with tunicamycin or gentamicin (Figure 4b). As expected from its activity as an ER stressor, tunicamycin induced CHOP in the nuclei of SGCs within 24 h. In contrast to its effect on hair cells, gentamicin increased the immunoreactivity to CHOP in SGCs, evident after 48 h of incubation.

### Gentamicin reduces the number of SGCs and synaptic ribbons but not hair cells in XBP1^+/−^ mice *in vivo*

In wild-type strains such as the CBA/J mouse, the outer hair cells (OHCs) are the primary target of chronic aminoglycoside ototoxicity *in vivo*^48^ and very little direct effect – if any – can be observed on SGCs. In view of the modest but significant gentamicin-induced CHOP expression in SGCs of cochlear explants, we investigated potential consequences of gentamicin-induced ER stress in a model of compromised UPR, an XBP1-haploinsufficient mouse. The local route of drug administration to the middle ear, chosen for this study, is able to isolate effects to the auditory periphery while avoiding adverse complications associated with systemic gentamicin treatment in the mouse.^30^ Preliminary experiments with a series of gentamicin concentrations starting at 0.09 M established 0.56 M as a suitable dose that caused a moderate auditory threshold shift while avoiding major pathophysiology (data not shown).

Surface preparations from XBP1^+/−^ and wild-type littermates locally treated with gentamicin *in vivo* were examined from base-to-apex 3 weeks after drug injection. OHCs were present in all parts of the cochlea in both wild-type and XBP1^+/−^ mice except for some scattered loss at the very end of the basal turn (Supplementary Figure S5c). Quantitation of hair cell loss along the entire cochlea confirmed only minor damage at the extreme, the basal turn with no difference between wild-type and XBP1^+/−^ mice.

**Figure 5:**
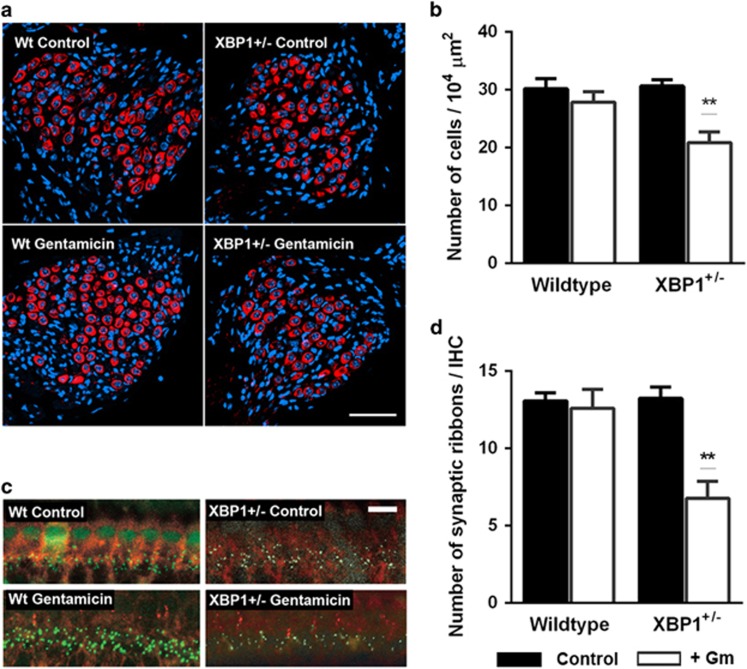
Gentamicin reduces the number of SGCs and IHC synapses in the basal turn of XBP1^+/−^ mice cochleae. Gentamicin (0.56 M) was locally injected into the middle ear through the bulla as described in the Materials and Methods section ‘Drug administration *in vivo*.' (**a** and **b**) Gentamicin reduces SGCs in XBP1^+/−^ but not in wild-type (XBP1^+/+^) littermates. (**a**) The number of SGCs was counted from high-magnification images of Rosenthal's canal of saline- or gentamicin-injected wild-type and XBP1^+/−^ mice. Red: neuronal class III *β*-tubulin staining for neural cells, blue: Hoechst 33342 staining for nuclei. The figure represents five different animals at each condition. Scale bar: 50 *μ*m. (**b**) Quantitative evaluations revealed that SGC density in the basal turn of XBP1^+/−^ mice but not in wild-type mice was significantly decreased by gentamicin. Filled bars, controls; open bars, gentamicin treatment. *n*=5 in each group; **P<0.01. Middle and apical turns were not affected. (**c** and **d**) Gentamicin reduces synaptic ribbons in XBP1^+/−^ but not in wild-type mice. (**c**) Hair cells were stained with anti-myo7 antibodies (red) and synaptic ribbons with antibodies to CtBP2 (green). The number of synaptic ribbons per IHC in the basal turn was quantified from 3-D images created by using Imaris software. Staining of some nuclei is consistent with a partial nuclear localization of CtBP2,^66^ which has been confirmed for IHCs.^67^ The figure represents three different animals at each condition. Scale bar: 20 *μ*m. (**d**) Quantitative evaluations demonstrated that synaptic ribbon density of XBP1^+/−^ mice but not of wild-type littermates was diminished by local injection of gentamicin. Filled bars, controls; open bars, gentamicin treatment. *n*=3 in each group; **P<0.01

In the absence of any discernible defects on hair cell integrity and prompted by the *in vitro* results, we then analyzed spiral ganglion density and synaptic connections. Three weeks after gentamicin injection, the SGCs were counted on mid-modiolar cryosections stained for *β*-tubulin and nuclei. There was a significant reduction in spiral ganglion density in the basal turn of the cochlea in XBP1^+/−^ mice but not in wild-type littermates (Figures 5a and b). The innervation of hair cells by the spiral ganglion was assessed by staining synaptic ribbons with antibody to CtBP2, a constituent of the ribbon protein RIBEYE. Gentamicin reduced the number of synaptic ribbons per IHC by approximately 50% in the basal turn of the cochlea of the XBP1^+/−^ mice but not of corresponding wild-type littermates (Figures 5c and d).

### Auditory physiology corroborates auditory pathology and ER stress

**Figure 6:**
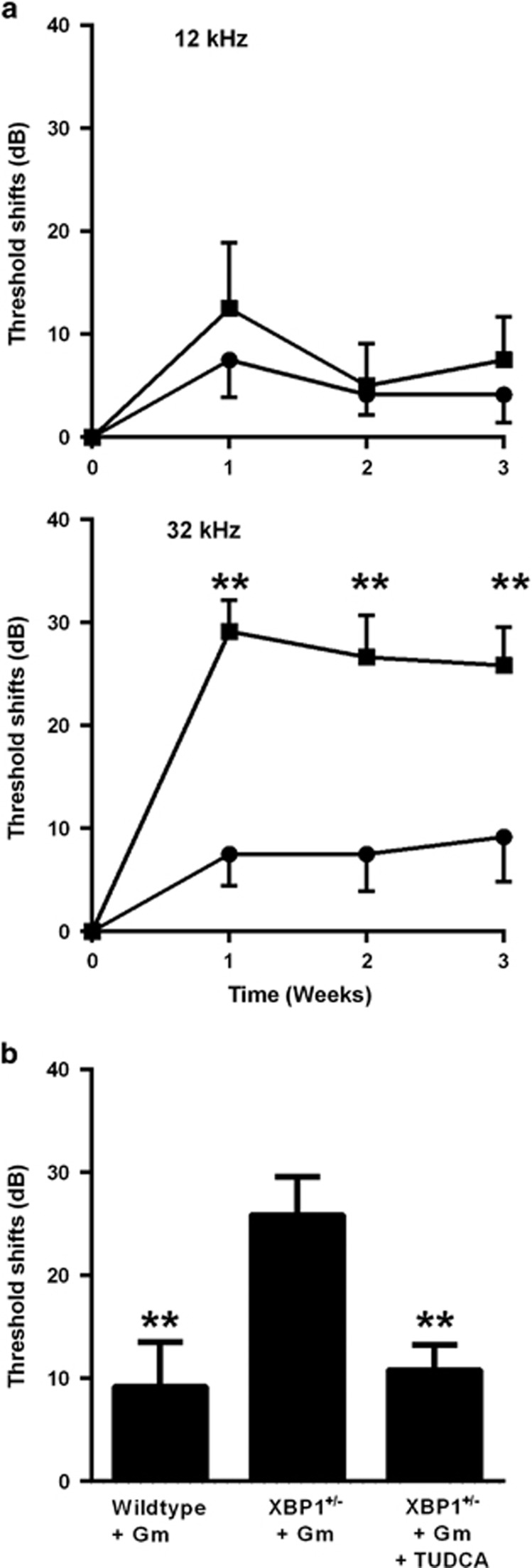
Auditory threshold shifts are induced by gentamicin and protected by TUDCA. (**a**) Gentamicin (0.56 M) was locally injected into the middle ear through the bulla as described in ‘Materials and Methods' section. Three weeks after treatment, large threshold shifts had developed at 32 kHz in XBP1^+/−^ mice (square symbols) but not in wild-type littermates (circles). Data are presented as mean+S.D. for XBP1^+/−^ mice and mean–S.D. for wild-types. *n*=6 in each group; **P<0.01. (**b**) TUDCA attenuates gentamicin ototoxicity in XBP1^+/−^ mice. Animals in all three groups received the local injection of gentamicin and, as indicated, TUDCA co-treatment (500 mg/kg sc.) at 6 days, 3 days and 3 h before gentamicin injection. Data are presented as means+S.D. of threshold shifts at 32 kHz, determined 3 weeks after treatment. *n*=6 in each group; **P<0.01

In order to assess the impact of the observed pathology on auditory function, we measured auditory brainstem responses (ABRs) and distortion product otoacoustic emissions (DPOAEs). ABR provides information on the ascending auditory pathway reflecting synaptic and neuronal activity, whereas DPOAE probes the functional integrity of OHCs. Deterioration of auditory thresholds was apparent 1 week after the injection of gentamicin and remained stable for up to 3 weeks, the latest time point studied (Figure 6a). Large threshold shifts were observed at 32 kHz in XBP1^+/−^ mice but not in wild-type littermates, which were little affected. Consistent with the morphological observations of intact OHCs, DPOAE remained unaffected by gentamicin treatment (Supplementary Figure S6).

Finally, in order to validate the potential contribution of protein misfolding to the gentamicin-induced changes in auditory thresholds, we treated animals with tauroursodeoxycholic acid (TUDCA), a clinically used chemical chaperone. Systemic TUDCA co-administration significantly attenuated gentamicin-induced ototoxicity in the XBP1^+/−^ mice (Figure 6b) as measured by ABR 3 weeks after the drug treatment.

## Discussion

Aminoglycoside-induced loss of translational fidelity in eukaryotes is evident from our experiments on HEK293 cells and, moreover, is clearly linked to the ribosomal activity of the drugs. The known misreading inducers geneticin and gentamicin, but not the non-misreading aminoglycoside hygromycin or the ribosomal translocation inhibitor cycloheximide, elicit a UPR. Gentamicin was selected as a classical clinical aminoglycoside to bridge the findings from our *in vitro* studies to the animal model. Geneticin was included because its inactivation by the APH(3′) enzyme allowed to control for the specificity of drug action.^39^ Modification of geneticin by APH(3′) (which abrogates its anti-ribosomal activity by phosphorylation of the 3′ OH group) indeed eliminated its ability to cause both misreading and ER stress. In contrast, APH(3′) did not affect the misreading activity of gentamicin, which lacks the 3′ OH group and thus is not a target for APH(3′).

We had primarily chosen HEK293 cells for study as these cells are readily transfected, facilitating the use of reporter constructs to study drug-induced misreading^49^ but similar results in human HeLa cells suggest the general nature of this response. The finding that the cell viability and the metabolic activity of HEK wild-type cells remain intact despite drug-induced mistranslation attests to the protective efficacy of cellular homeostatic responses such as the UPR and allows us to extrapolate that the UPR, at least in part, mitigates mistranslation induced by aminoglycosides in eukaryotic organisms.

Consistent with this notion, XBP1^+/−^ haploinsufficient mice but not wild-type mice sustain gentamicin-induced loss of SGCs. XBP1 is one of the central components in the three canonical pathways of the UPR, regulating molecular chaperones and promoting ER-associated degradation.^50^ The crucial function of XBP1 for cell survival is evident from the embryonic lethality of homozygous XBP1 knock-out mice.^37^ Haploinsufficient mice are viable but are less capable of inducing chaperones and promoting ERAD under ER stress conditions.^51^ Consequently, ER stress is prone to damage cells in XBP1^+/−^ but not in wild-type mice.

Aminoglycoside-induced death of hair cells has mostly been associated with inhibition of host-cell protein synthesis^47, 52^ and oxidative stress,^48^ and evidence for an involvement of ER stress in ototoxicity has been indirect or lacking. Upregulation of heat shock proteins protects the mouse inner ear in part from aminoglycoside-induced ototoxicity *in vivo*.^53^ However, the ER stress marker m-calpain is unaffected by aminoglycoside treatment in the mouse cochlea *in vivo*.^54^ We show here that no ER stress marker develops in hair cells despite the extensive damage that gentamicin causes in cochlear explants. This is in contrast to the action of the ER stressor tunicamycin, a finding consistent with previous observations of tunicamycin-induced hearing loss in the rat.^55^ Further distinguishing the pathological mechanisms of the two drugs, the hair cell loss caused by tunicamycin broadly encompasses all regions of the cochlea, whereas the pattern of gentamicin-induced damage in the explants follows the base-to-apex gradient characteristic of aminoglycosides.^48^

Our results clarify that aminoglycosides can induce ER stress in mammalian tissues, including the inner ear. In agreement with our results, markers of ER stress have also been observed in rat kidneys as part of the nephrotoxic actions of gentamicin treatment.^56^ In the cochlea, drug-induced ER stress is limited to neurons of the spiral ganglion. In the *in vivo* model presented here, a single low dose of gentamicin does not lead to hair cell death. However, SGCs were significantly reduced in the base of the cochlea, corroborating the *in vitro* results on ER stress in the nerve but not in hair cells. In accord with a decreased density of SGCs, synaptic connections to hair cells are lost, providing an explanation for the observed high-frequency threshold shift. It is interesting in this context that a loss of afferent nerve terminals and subsequent degeneration of the cochlear nerve has also been observed after moderate noise exposure that leaves the sensory cells intact.^57^

On a mechanistic level, disruption of translational fidelity causes protein misfolding and aggregation. The ability of XBP1 to maintain cell integrity upon drug-induced mistranslation appears to be mediated by induction of ER chaperones such as BiP, which we find to be upregulated. Consequently, the selective actions of gentamicin on SGCs and synapses suggest a heightened sensitivity to neurodegeneration in the XBP1^+/−^ haploinsufficient mice. Although our results imply an important role for XBP1, additional investigations are required to address its exact role in aminoglycoside-induced ER stress, given the frequent intercalation of the three branches of the UPR.^12, 50^

Protein misfolding has been associated with a variety of disorders collectively termed conformational diseases.^58^ Presumably, cell-type-specific differences in the buffering capacity of the proteostasis network account for the cell or organ selectivity in some of these diseases.^59^ The hypothesis presented here that loss of SGCs in XBP1^+/−^ haploinsufficient mice is conferred by the drug's misreading activity is supported *in vivo* by the observation that administration of a chemical chaperone significantly alleviated the gentamicin-induced hearing loss. Specifically, we postulate that the UPR is normally able to maintain a protein folding equilibrium in the presence of aminoglycoside-induced mistranslation in SGCs. However, when the UPR system is compromised, for example, by genetic haploinsufficiency of XBP1, aminoglycoside-induced mistranslation can manifest as neuropathology.

## Materials and Methods

### Materials and sources

Mouse monoclonal anti-GADD 153 (B-3) antibody (Santa Cruz Biotechnology, Dallas, TX, USA); polyclonal antibody against neuronal class III *β*-tubulin (Covance, Princeton, NJ, USA); monoclonal anti-CtBP2 antibody (BD Biosciences, San Jose, CA, USA); polyclonal antibody against p-eIF2*α* (Cell Signaling, Danvers, MA, USA); polyclonal anti-myosin 7a antibody (Proteus Biosciences, Ramona, CA, USA); secondary goat anti-rabbit antibody conjugated with Texas Red (Abcam, Cambridge, MA, USA); rhodamine phalloidin, Invitrogen (Life Technologies, Carlsbad, CA, USA); HEK293 cells (Innoprot, Biscay, Spain); geneticin, gentamicin, tunicamycin, cycloheximide, arsenite, saponin and HEK *aph*(3′) cells (Sigma Aldrich, St. Louis, MO, USA); hygromycin (PAA Laboratories, Cansera, Canada); nucleotide primers (Microsynth, Balgach, Switzerland); cell culture media and trypsin (Life Technologies).

### Assessment of mistranslation

Misreading and stop codon readthrough were assessed in gain-of-function dual-luciferase assays.^60, 61^ For translation in RRLs (Promega, Madison, WI, USA), luciferase mRNA was produced *in vitro* using T7 RNA polymerase (Thermo Scientific, Waltham, MA, USA) and plasmids pGL4.14 (humanized firefly luciferase, hFluc) and pGL4.75 (humanized renilla luciferase, hRluc; both from Promega), where the mammalian promoter was replaced by the T7 bacteriophage promoter. For misreading, we replaced residue His245 (CAC codon) with Arg245 (CGC near-cognate) in the hFluc protein by site-directed mutagenesis. Readthrough was assessed with a fusion construct in which hRluc and hFluc were fused by a 27-nucleotide linker encoding the polypeptide STCDQPFGF, using overlap PCR mutagenesis to result in the pT7 hRluc-hFluc vector; a UGA nonsense-codon was introduced at the glutamine residue (wild-type CAA) of the linker sequence by site-directed PCR mutagenesis. A cell-free luciferase translation assay was performed as described.^60^

Mistranslation in HEK cells was determined using the pRM hRluc-hFluc H245R vector, where His245 (CAC codon) was replaced by Arg245 (CGC codon) in the pRM hRluc-hFluc vector. Readthrough was determined by pRM hRluc-hFluc D357X, where Asp357 (GAC codon) was replaced by a UGA nonsense-codon in the firefly luciferase transcript. Both constructs were designed by site-directed PCR mutagenesis. HEK wild-type cells were transfected with reporter plasmid using TurboFect (Fermentas, Vilnius, Lithuania) according to the manufacturer‘s protocol. After a 24-h incubation, medium was replaced by F10 with 15 *μ*g/ml saponin. Aminoglycoside antibiotics were added and cells were incubated for another 24 h. Cells were lysed and luciferase activities determined; hRluc mRNA was used as an internal control and misreading and readthrough were quantified by calculating mutant firefly/renilla activities. The basal error frequency of the eukaryotic ribosome^62^ is 4 × 10^–^^4^ to 10^–^^5^. For each set of replicates, the hFluc/hRluc ratio of the untreated samples were set as 1, which reflects this basal error frequency. Luminescence was measured in a luminometer FLx800 (Bio-Tek Instruments, Winooski, VT, USA).

### Viability assay

HEK cells were grown to 70% confluence and treated for the indicated time with 16 *μ*M geneticin or 400 *μ*M gentamicin in F10 medium with 15 *μ*g/ml saponin. Ten percent Alamar Blue solution was added (v/v) for 3 h and fluorescence was monitored at 530 nm for excitation and 590 nm for emission. The fluorescence level of the control sample (untreated) was set as 100% after subtraction of background fluorescence, measured in cell-free wells.

### Sytox dead cell stain

HEK cells were grown to 60% confluence in DMEM with 10% fetal bovine serum (FBS). Medium was changed to F10 with 15 *μ*g/ml saponin and aminoglycoside antibiotics were added and cells were incubated for 24 or 48 h. Cells were detached by adding 100 μl accutase (Life Technologies) and were resuspended in 400 *μ*l FACS buffer (1x phosphate-buffered saline (PBS), 2% FBS) and transferred to FACS tubes. Sytox Red (Life Technologies) was added to the cell suspension. The nucleic acid stain penetrates cells with compromised plasma membranes but will not cross uncompromised cell membranes. The samples were then analyzed with a BD FACS Canto II (BD Biosciences, Allschwil, Switzerland) and the FlowJo data analysis software (FlowJo, Ashland, OR, USA).

### Microarray analysis

See the legend of Supplementary Figure S2.

### Proteome analysis

Cell samples were incubated with lysis buffer (150 mM NaCl, 0.1% SDS, 0.5% Na-deoxycholate, 50 mM Tris pH 7.5 and 1 × complete protease inhibitor (Roche, Rotkreuz, Switzerland)) for 10 min at room temperature (RT) on a shaking mixer. The lysate was ultrasonicated for 10 min and centrifuged for 20 min at 16 000 × *g* at 4 °C. Eighty micrograms of protein of each sample were used for iTRAQ labeling (AB SCIEX, Framingham, MA, USA).

Each iTRAQ 4-plex experiment was carried out with two biological replicates of untreated HEK wild-type cells (114 and 116 label) and two biological replicates of cells treated with 16 *μ*M geneticin for 32 h (115 and 117 label) following the manufacturer's protocol. iTRAQ samples were pooled, dried, reconstituted in solvent A (5% ACN, 8 mM KH_2_PO_4_ and pH 4.5), and fractionated by HILIC-HPLC (Pack Polyamine II, 250 × 4 mm, 120 Å S-5 *μ*m, YMC). The column was equilibrated with solvent A. Peptides were eluted using solvent B (5% ACN, 100 mM KH_2_PO_4_ and pH 4.5) by a gradient of: 0–7.5 min, 0% B; 7.5–37.5 min, 0–50% B; 37.5–42.5 min, 50–100% B; 42.5–47.5 min, 100% B at a flow rate of 0.4 ml/min. The resulting 13 fractions were desalted using ZipTips (Millipore, Billerica, MA, USA) according to the manufacturer's protocol and reconstituted in solvent C (3% ACN and 0.1% formic acid) for LC-MS/MS analysis. Samples were auto-injected into an Eksigent-nano-HPLC system and separated on a custom reverse phase tip column (75 *μ*m × 150 mm) packed with C_18_ material (3 *μ*m, 200 Å, AQ, Bischoff GmbH, Leonberg, Germany). The column was equilibrated with solvent C and 5% solvent D (0.2% FA in ACN). For elution, a flow rate of 300 nl/min was used and a gradient of 0–70 min, 5–25% D; 70–85 min, 25–50% D; 85–88 min, 50–98% D. High accuracy mass spectra were acquired with an AB SCIEX 5600 mass spectrometer (AB SCIEX) in the range of 385–1250 m/z. Up to 36 data-dependent MS/MS were recorded in high sensitivity mode of the most intense ions with charge states 2+, 3+ and 4+ using collision-induced dissociation. Target ions already selected for MS/MS were dynamically excluded for 90 s after three occurrences. MS/MS data were analyzed using Mascot 2.4 (Matrix Science, Boston, MA, USA) and searched against a decoyed human database from Swissprot (Lausanne, Switzerland; release December 2012) concatenated with an in-house build contaminant database. The search parameters were: precursor ion mass tolerance of 20 p.p.m., fragment ion mass tolerance of 0.05 Da, trypsin digestion, fixed modifications of MMTS-labeled cysteine, 4-plex iTRAQ modifications of free amines at the N-termini and of lysine and variable modification 4-plex iTRAQ of tyrosine. Peptides without 4-plex iTRAQ labeling at the N-terminus or at a lysine were excluded from the analysis. Scaffold_4.1 (Proteome Software Inc., Portland, OR, USA) was used to validate MS/MS-based peptide and protein identifications. We identified and quantified 1785 proteins (protein prophet probability 95%, minimum two peptides for identification of a protein and minimum Mascot Ionscore of 40). After the permutation test and further amendment of the *P*-value with the Bonferroni correction, 77 proteins were found to be regulated (*P*-value<0.05). Thirty-five proteins were upregulated based on a threshold of 0.3 (log_2_ scale).

### XBP1-splicing assay and qPCR

RNA samples from HEK cells were prepared using Trizol extraction (Life Technologies) and were reverse transcribed using a ThermoScript RT-PCR System (Life Technologies) according to the manufacturer's instructions. The XBP1-splicing assay used XBP1-specific primers that amplify spliced (−26 nt) and unspliced XBP1 mRNA (forward 5′-TTACGAGAGAAAACTCATGGCC-3′, reverse 5′-GGGTCCAAGTTGTCCAGAATGC-3′). PCR products were analyzed on a 2.7% agarose gel. Amplification of glyceraldehyde 3-phosphate dehydrogenase (GAPDH) cDNA served as loading control. For qPCR the Quantitect SYBR Green PCR Kit (Qiagen, Hilden, Germany) was used together with the 7500 Fast Real-Time PCR System (Applied Biosystems, Zug, Switzerland). The primers were CHOP: forward 5′-GCGCATGAAGGAGAAAGAAC-3′, reverse 5′-CCAATTGTTCATGCTTGGTG-3′ BiP: forward 5′-TTTCTGCCATGGTTCTCACTAAAA-3′, reverse 5′-AACATTTAGGCCAGCAATAGTTCC-3′ GAPDH: forward 5′-ACCCACTCCTCCACCTTTGA-3′, reverse 5′-CTGTTGCTGTAGCCAAATTCGT-3′ GRP94 forward 5′-TGGGAAGAGGTTCCAGAATG-3′, reverse 5′-GTTGCCAGACCATCCGTACT-3′. For relative quantification, GAPDH mRNA served as a reference. Measured quantification cycles were analyzed according to Pfaffl,^63^ comparing treated with untreated samples. Three biological replicates were run in triplicates each and means and standard deviations were calculated.

### Western blot

HEK cells were grown to 60% confluence in DMEM with 10% FBS. Medium was changed to F10 with 15 *μ*g/ml saponin and aminoglycoside antibiotics were added and cells were incubated for 24 h. Cells were lysed in hypotonic buffer (20 mM HEPES pH 7.5, 10 mM KCl, 3 mM Mg acetate, 1 mM DTT and 10 *μ*g/ml DNase I) and ultrasonicated. Lysates were centrifuged (13 000 r.p.m., 10 min) and protein concentration in the supernatant was measured by the Micro BCA Protein Assay Kit (Thermo Scientific). Ten micrograms of total protein were resolved on 10% SDS-polyacrylamide gels and blotted on nitrocellulose membranes, which were probed with specific antibodies. Amersham ECL Prime Western Blotting Detection Reagent (RPN2232; GE Healthcare, Glattbrugg, Switzerland) was used as a substrate for the horseradish peroxidase (HRP). The specific antibodies used in this study were: anti-BiP antibody (Abcam, ab21685); anti-GRP94 antibody (Abcam, ab87886); anti-ATF4 antibody (Abcam, ab23760); anti-*β*-actin antibody (A1978-200UL; Sigma-Aldrich); HRP-conjugated goat anti-rabbit (Invitrogen, G-21234) and goat anti-mouse antibodies (Invitrogen, A10551).

### UPR reporter assay

Reporter plasmids UPRE (p5xATF6-GL3)^44^ and ERSE (pGL3-GRP78P(-132)-luc)^45^ carrying luciferase under the control of UPR-specific cis-acting elements were kind gifts from Kazutoshi Mori (Kyoto University, Japan). HEK cells were grown to 60% confluence and co-transfected with reporter constructs and pGL4.75 (Rluc) using TurboFect reagent (Fermentas) according to the manufacturer's protocol. After a 24-h incubation, medium was replaced by F10 with 15 μg/ml saponin. Aminoglycosides were added and cells were incubated for another 24 h. Cells were lysed and luciferase activities determined. Normalization of luciferase activities was performed as described above. Cycloheximide was used as a mistranslation negative control and tunicamycin was used as a positive control for UPR.

### P-eIF2*α* immunofluorescence assay

HEK cells grown on poly-D-lysine (Sigma Aldrich)-coated cover slips (Thermo Scientific) were treated for 24 h with geneticin in F10 medium with 15 *μ*g/ml saponin, or with arsenite for 1 h (positive control). Cells were then fixed with 4% paraformaldehyde and methanol and incubated with blocking solution (1 × PBS with 1% BSA and 0.5% saponin) for 1 h at RT. Immunostaining used a rabbit polyclonal antibody against p-eIF2*α* (1 : 250) and a secondary goat anti-rabbit antibody conjugated with Texas Red (1 : 250) diluted in blocking solution. Cover slips were mounted on glass slides (VWR) using Dapi fluoromount (Southern Biotech, Birmingham, AL, USA), and cells were imaged using a Lyca Sp2 confocal microscope and a 63 × objective. p-eIF2*α* and nuclear signals were quantified using Imaris software (Bitplane, Belfast, UK) and the dots-per-cell ratio was calculated.

### Animals

Male and female CBA/J mice were purchased from Harlan Sprague-Dawley Co. (http://www.harlan.com/products_and_services/research_models_and_services/research_models/cbaj_inbred_mice.hl) at an age of 6–8 weeks and bred in-house in order to obtain pups for organotypic cultures of the post-natal organ of Corti and SGCs (see next section). XBP1^+/−^ mice were from a stock kindly provided by Dr. Laurie H Glimcher and received via Dr. Randal J Kaufman, University of Michigan. Littermates served as wild-type (XBP1^+/+^) controls. Animals were kept on a 12-h light/12-h dark cycle with free access to water and diet (Purina 5001, Lab Diet, St. Louis, MO, USA) and used in the *in vivo* studies at an age of 3–4 months. Experimental protocols were approved by the University of Michigan Committee on the Use and Care of Animals and animal care was under the supervision of the University of Michigan's Unit for Laboratory Animal Medicine.

### Organotypic cultures of post-natal organ of Corti and SGCs

The procedures were as described previously.^64^ Mice at post-natal day 2–3 (p2–3) were killed and cochleae dissected in cold Hank's Balanced Salt Solution to isolate the organ of Corti; SGCs were dissected from the base to the middle of the modiolus. Explants were placed onto a culture dish in 2 ml of medium consisting of basal medium Eagle, 1% serum-free supplement (Gibco #51500-056, Life Technologies, Grand Island, NY, USA), 1% bovine serum albumin, 5 mg/ml glucose and 10 U/ml penicillin G, allowed to settle for 4 h (37 °C, 5% CO_2_) and incubated for 2 days to mitigate dissection stress. Medium was then exchanged for new media with or without drugs and incubation continued. For immunofluorescent labeling, explants were fixed with 4% paraformaldehyde overnight at 4 °C and permeabilized for 30 min with 3% Triton X-100 in PBS at RT. After three washes with PBS and blocking with 10% goat serum for 30 min at RT, incubation with the primary antibodies followed at 4 °C for 72 h. After three washes with PBS, secondary antibodies were applied (Alexa Fluor 488-conjugated goat anti-mouse and Alexa Fluor 546-conjugated goat anti-rabbit antibody; 1 : 200 in PBS) at 4 °C overnight in darkness. After several rinses, specimens were mounted on a slide with Prolong Gold anti-fade reagent (Life Technologies) and imaged with an Olympus Fluoview Confocal Laser Scanning Microscope-FV500 (Olympus America, Center Valley, PA, USA). For staining of hair cells, specimens were incubated at RT with rhodamine phalloidin (1 : 100) for 1 h; or for staining of nuclei with Hoechst 33342 (2 *μ*g/ml in PBS) for 40 min. Presence or absence of OHCs and IHCs was determined on a Leitz Orthoplan microscope (Leica, Wetzlar, Germany) whose right objective had a 0.19-mm scale imposed on the field. Successive 0.19-mm fields were evaluated beginning at the apex by observers blinded to the experimental conditions. Cell counts were compared with a normative database (KHRI Cytocochleogram, version 3.0.6, Kresge Hearing Research Institute, University of Michigan, Ann Arbor, MI, USA).

### Drug administration *in vivo*

Gentamicin was locally delivered as previously described.^65^ Mice were anesthetized with an intraperitoneal injection of xylazine (7 mg/kg) and ketamine (90 mg/kg) and body temperature was maintained. The temporal bone was approached via a retro-auricular incision and a small hole was made in the thin part of the bulla with a 30-G needle. Surgical tubing was inserted through the hole, and 10 *μ*l of 0.56 M gentamicin dissolved in saline was slowly injected. The skin incision was closed with tissue adhesive. TUDCA (Calbiochem, EMD Millipore) was dissolved in 0.15 M NaHCO_3_ (adjusted to pH 7.4) and injected subcutaneously at 500 mg/kg body weight 6 days, 3 days and 3 h before gentamicin administration. Injections of 0.15 M NaHCO_3_ served as vehicle controls. Injection of TUDCA did not cause any apparent side effects.

### Hair cell counts in adult mice

Three weeks after injections, cochleae were fixed as described above. The apical bony capsule was removed and the cochlea decalcified in 4% sodium EDTA (adjusted to pH 7.4) for 7 days at 4 °C. Subsequently, cochleae were dissected into apical, middle and basal segments. Segments were permeabilized in 3% Triton X-100 for 30 min at RT, washed three times with PBS and incubated with rhodamine phalloidin (1 : 100) at RT for 1 h. The procedures for cell counting were the same as for explants.

### Quantification of SGCs and synaptic ribbons

Following decalcification with 4% EDTA, cochleae were cryo-sectioned. Sections of 8 *μ*m thickness were incubated in 0.3% Triton X-100 in PBS for 30 min at RT, blocked with 10% goat serum for 30 min, followed by incubation with anti-neuronal class III *β*-tubulin antibody (1 : 2000) for 48 h at 4 °C. After three rinses in PBS, sections were incubated with a secondary antibody (Alexa Fluor 546-conjugated; 1 : 500) at 4 °C overnight in darkness. After three washes with PBS, sections were stained with Hoechst 33342 (2 *μ*g/ml in PBS) for 40 min at RT. After a final wash, the slides were mounted with Prolong Gold anti-fade reagent. Controls were processed without primary antibody. The number of SGCs in Rosenthal's canal was quantified using ImageJ software (National Institutes of Health, Bethesda, MD, USA) by counting the *β*-tubulin- and Hoechst-positive cells on images taken with an Olympus laser confocal microscope. Two mid-modiolar sections, separated by 40–50 *μ*m, were used to obtain the average for each animal. For synaptic ribbon counts, cryosections were incubated for 30 min at RT with 5% donkey serum in PBS with 0.3% Triton X-100 and overnight in darkness at 4 °C with antibodies against CtBP2 (1 : 200) and Myo7a (1 : 200). After three washes in PBS (15 min each), tissues were incubated with secondary antibodies (Alexa Fluor 488- and Alexa Fluor 546-conjugated; 1 : 1000) at RT for 1 h. After three washes, the epithelia were mounted and images taken on an Olympus laser confocal microscope. Images were reconstructed three-dimensionally using Imaris software (Bitplane). The number of synaptic ribbons was quantified per IHC based on an average of 14 IHCs per sample.

### Auditory function measurements

For ABRs, animals were anesthetized with an intraperitoneal injection of xylazine (7 mg/kg), ketamine (65 mg/kg) and acepromazine (2 mg/kg), and placed in a sound-isolated and electrically shielded booth (Acoustic Systems, Austin, TX, USA). Body temperature was maintained near 37 °C with a heating pad. Acoustic stimuli were delivered monaurally to a Beyer earphone attached to a speculum inserted into the left ear canal. Subdermal electrodes were inserted at the vertex of the skull, under the left ear and under the right ear (ground). ABRs were measured at 12 and 32 kHz using Tucker Davis Technology (TDT) System III hardware and SigGen/Biosig software (Tucker Davis Technology, Alachua, FL, USA) to present the stimuli (15 ms tone bursts, 1 ms rise-fall time) and record up to 1024 responses for each stimulus level. Thresholds were determined by reducing the intensity in 10-dB increments and then in 5-dB steps until no organized responses were detected. Threshold shifts were calculated for individual animals as the difference in auditory thresholds between ABR measurements before and at the end of the studies. For the DPOAE procedure, see the legend of Supplementary Figure S6.

### Statistical analysis

Data were evaluated by one-way ANOVA followed by Tukey's honestly significant difference tests using JMP version 8.0.1 (SAS Institute Inc., Cary, NC, USA) or Student's *t*-test. All tests were two-sided with significance set at *P*<0.05.

## Abbreviations

APH(3'): aminoglycoside phosphotransferase
BiP: binding immunoglobulin protein
CHOP: C/EBP homologous protein
DPOAE: distortion product otoacoustic emissions
ER: endoplasmic reticulum
ERAD: ER-associated degradation
FBS: fetal bovine serum
GAPDH: glyceraldehyde 3-phosphate dehydrogenase
GRP94: glucose-regulated protein 94
hRluc: humanized renilla luciferase
hFluc: humanized firefly luciferase
IHCs: inner hair cells
OHCs: outer hair cells
p-eIF2*α*: phosphorylated eukaryotic initiation factor 2 alpha
PBS: phosphate-buffered saline
RRL: rabbit reticulocyte lysate
RT: room temperature
SGCs: spiral ganglion cells
TUDCA: tauroursodeoxycholic acid
UPR: unfolded protein response
XBP1: X-box binding protein-1

## References

[bib1] Drummond DA, Wilke CO. Mistranslation-induced protein misfolding as a dominant constraint on coding-sequence evolution. Cell 2008; 134: 341–352. 18662548 10.1016/j.cell.2008.05.042 PMC2696314

[bib2] Goltermann L, Good L, Bentin T. Chaperonins fight aminoglycoside-induced protein misfolding and promote short-term tolerance in Escherichia coli. J Biol Chem 2013; 288: 10483–10489. 23447537 10.1074/jbc.M112.420380 PMC3624430

[bib3] Prostko CR, Brostrom MA, Malara EM, Brostrom CO. Phosphorylation of eukaryotic initiation factor (eIF) 2 alpha and inhibition of eIF-2B in GH3 pituitary cells by perturbants of early protein processing that induce GRP78. J Biol Chem 1992; 267: 16751–16754. 1512215

[bib4] Shi Y, Vattem KM, Sood R, An J, Liang J, Stramm L et al. Identification and characterization of pancreatic eukaryotic initiation factor 2 alpha-subunit kinase, PEK, involved in translational control. Mol Cell Biol 1998; 18: 7499–7509. 9819435 10.1128/mcb.18.12.7499 PMC109330

[bib5] Tirasophon W, Welihinda AA, Kaufman RJ. A stress response pathway from the endoplasmic reticulum to the nucleus requires a novel bifunctional protein kinase/endoribonuclease (Ire1p) in mammalian cells. Genes Dev 1998; 12: 1812–1824. 9637683 10.1101/gad.12.12.1812 PMC316900

[bib6] Haze K, Yoshida H, Yanagi H, Yura T, Mori K. Mammalian transcription factor ATF6 is synthesized as a transmembrane protein and activated by proteolysis in response to endoplasmic reticulum stress. Mol Biol Cell 1999; 10: 3787–3799. 10564271 10.1091/mbc.10.11.3787 PMC25679

[bib7] Yoshida H, Okada T, Haze K, Yanagi H, Yura T, Negishi M et al. ATF6 activated by proteolysis binds in the presence of NF-Y (CBF) directly to the cis-acting element responsible for the mammalian unfolded protein response. Mol Cell Biol 2000; 20: 6755–6767. 10958673 10.1128/mcb.20.18.6755-6767.2000 PMC86199

[bib8] Shen J, Chen X, Hendershot L, Prywes R. ER stress regulation of ATF6 localization by dissociation of BiP/GRP78 binding and unmasking of Golgi localization signals. Dev Cell 2002; 3: 99–111. 12110171 10.1016/s1534-5807(02)00203-4

[bib9] Bertolotti A, Zhang Y, Hendershot LM, Harding HP, Ron D. Dynamic interaction of BiP and ER stress transducers in the unfolded-protein response. Nat Cell Biol 2000; 2: 326–332. 10854322 10.1038/35014014

[bib10] Schroder M, Kaufman RJ. The mammalian unfolded protein response. Annu Rev Biochem 2005; 74: 739–789. 15952902 10.1146/annurev.biochem.73.011303.074134

[bib11] Zhang K, Kaufman RJ. The unfolded protein response: a stress signaling pathway critical for health and disease. Neurology 2006; 66: S102–S109. 16432136 10.1212/01.wnl.0000192306.98198.ec

[bib12] Hetz C. The unfolded protein response: controlling cell fate decisions under ER stress and beyond. Nat Rev Mol Cell Biol 2012; 13: 89–102. 22251901 10.1038/nrm3270

[bib13] Travers KJ, Patil CK, Wodicka L, Lockhart DJ, Weissman JS, Walter P. Functional and genomic analyses reveal an essential coordination between the unfolded protein response and ER-associated degradation. Cell 2000; 101: 249–258. 10847680 10.1016/s0092-8674(00)80835-1

[bib14] Yoshida H, Matsui T, Hosokawa N, Kaufman RJ, Nagata K, Mori K. A time-dependent phase shift in the mammalian unfolded protein response. Dev Cell 2003; 4: 265–271. 12586069 10.1016/s1534-5807(03)00022-4

[bib15] Lin JH, Li H, Yasumura D, Cohen HR, Zhang C, Panning B et al. IRE1 signaling affects cell fate during the unfolded protein response. Science 2007; 318: 944–949. 17991856 10.1126/science.1146361 PMC3670588

[bib16] Haynes CM, Titus EA, Cooper AA. Degradation of misfolded proteins prevents ER derived oxidative stress and cell death. Mol Cell 2004; 15: 767–776. 15350220 10.1016/j.molcel.2004.08.025

[bib17] Edelmann P, Gallant J. Mistranslation in *E. coli*. Cell 1977; 10: 131–137. 138485 10.1016/0092-8674(77)90147-7

[bib18] Palmer E, Wilhelm JM. Mistranslation in a eucaryotic organism. Cell 1978; 13: 329–334. 75070 10.1016/0092-8674(78)90201-5

[bib19] Palmer E, Wilhelm JM, Sherman F. Phenotypic suppression of nonsense mutants in yeast by aminoglycoside antibiotics. Nature 1979; 277: 148–150. 366439 10.1038/277148a0

[bib20] Stansfield I, Jones KM, Herbert P, Lewendon A, Shaw WV, Tuite MF. Missense translation errors in Saccharomyces cerevisiae. J Mol Biol 1998; 282: 13–24. 9733638 10.1006/jmbi.1998.1976

[bib21] Wilhelm JM, Pettitt SE, Jessop JJ. Aminoglycoside antibiotics and eukaryotic protein synthesis: structure—function relationships in the stimulation of misreading with a wheat embryo system. Biochem 1978; 17: 1143–1149. 656378 10.1021/bi00600a001

[bib22] Buchanan JH, Stevens A, Sidhu J. Aminoglycoside antibiotic treatment of human fibroblasts: intracellular accumulation, molecular changes and the loss of ribosomal accuracy. Eur J Cell Biol 1987; 43: 141–147. 3569303

[bib23] Abraham AK, Pihl A. Effect of protein synthesis inhibitors on the fidelity of translation in eukaryotic systems. Biochim Biophys Acta 1983; 741: 197–203. 6652088 10.1016/0167-4781(83)90059-3

[bib24] Burke JF, Mogg AE. Suppression of a nonsense mutation in mammalian cells in vivo by the aminoglycoside antibiotics G-418 and paromomycin. Nucleic Acids Res 1985; 13: 6265–6272. 2995924 10.1093/nar/13.17.6265 PMC321951

[bib25] Howard M, Frizzell RA, Bedwell DM. Aminoglycoside antibiotics restore CFTR function by overcoming premature stop mutations. Nat Med 1996; 2: 467–469. 8597960 10.1038/nm0496-467

[bib26] Bedwell DM, Kaenjak A, Benos DJ, Bebok Z, Bubien JK, Hong J et al. Suppression of a CFTR premature stop mutation in a bronchial epithelial cell line. Nat Med 1997; 3: 1280–1284. 9359706 10.1038/nm1197-1280

[bib27] Nudelman I, Rebibo-Sabbah A, Shallom-Shezifi D, Hainrichson M, Stahl I, Ben-Yosef T et al. Redesign of aminoglycosides for treatment of human genetic diseases caused by premature stop mutations. Bioorg Med Chem Lett 2006; 16: 6310–6315. 16997553 10.1016/j.bmcl.2006.09.013

[bib28] Jin QH, Zhao B, Zhang XJ. Cytochrome c release and endoplasmic reticulum stress are involved in caspase-dependent apoptosis induced by G418. Cell Mol Life Sci 2004; 61: 1816–1825. 15241557 10.1007/s00018-004-4143-7 PMC11138620

[bib29] Quiros Y, Vicente-Vicente L, Morales AI, López-Novoa JM, López-Hernández FJ. An integrative overview on the mechanisms underlying the renal tubular cytotoxicity of gentamicin. Toxicol Sci 2011; 119: 245–256. 20829429 10.1093/toxsci/kfq267

[bib30] Forge A, Schacht J. Aminoglycoside antibiotics. Audiol Neurootol 2000; 5: 3–22. 10686428 10.1159/000013861

[bib31] Kass JS, Shandera WX. Nervous system effects of antituberculosis therapy. CNS drugs 2010; 24: 655–667. 20658798 10.2165/11534340-000000000-00000

[bib32] Tan J, Shepherd RK. Aminoglycoside-induced degeneration of adult spiral ganglion neurons involves differential modulation of tyrosine kinase B and p75 neurotrophin receptor signaling. Am J Pathol 2006; 169: 528–543. 16877354 10.2353/ajpath.2006.060122 PMC1780161

[bib33] Jeong SW, Kim LS, Hur D, Bae WY, Kim JR, Lee JH. Gentamicin-induced spiral ganglion cell death: apoptosis mediated by ROS and the JNK signaling pathway. Acta Otolaryngol 2010; 130: 670–678. 20082569 10.3109/00016480903428200

[bib34] Hinojosa R, Lerner SA. Cochlear neural degeneration without hair cell loss in two patients with aminoglycoside ototoxicity. J Infect Dis 1987; 156: 449–455. 3611831 10.1093/infdis/156.3.449

[bib35] Sone M, Schachern PA, Paparella MM. Loss of spiral ganglion cells as primary manifestation of aminoglycoside ototoxicity. Hear Res 1998; 115: 217–223. 9472750 10.1016/s0378-5955(97)00191-3

[bib36] Wu WJ, Sha SH, McLaren JD, Kawamoto K, Raphael Y, Schacht J. Aminoglycoside ototoxicity in adult CBA, C57BL and BALB mice and the Sprague-Dawley rat. Hear Res 2001; 158: 165–178. 11506949 10.1016/s0378-5955(01)00303-3

[bib37] Reimold AM, Etkin A, Clauss I, Perkins A, Friend DS, Zhang J et al. An essential role in liver development for transcription factor XBP-1. Genes Dev 2000; 14: 152–157. 10652269 PMC316338

[bib38] Southern PJ, Berg P. Transformation of mammalian cells to antibiotic resistance with a bacterial gene under control of the SV40 early region promoter. J Mol Appl Genet 1982; 1: 327–341. 6286831

[bib39] Takano M, Okuda M, Yasuhara M, Hori R. Cellular toxicity of aminoglycoside antibiotics in G418-sensitive and -resistant LLC-PK1 cells. Pharm Res 1994; 11: 609–615. 8058626 10.1023/a:1018999423464

[bib40] Kampinga HH, Hageman J, Vos MJ, Kubota H, Tanguay RM, Bruford EA et al. Guidelines for the nomenclature of the human heat shock proteins. Cell Stress Chaperones 2009; 14: 105–111. 18663603 10.1007/s12192-008-0068-7 PMC2673902

[bib41] Harding HP, Novoa I, Zhang Y, Zeng H, Wek R, Schapira M, Ron D. Regulated translation initiation controls stress-induced gene expression in mammalian cells. Mol Cell 2000; 6: 1099–1108. 11106749 10.1016/s1097-2765(00)00108-8

[bib42] Manuvakhova M, Keeling K, Bedwell DM. Aminoglycoside antibiotics mediate context dependent suppression of termination codons in a mammalian translation system. RNA 2000; 6: 1044–1055. 10917599 10.1017/s1355838200000716 PMC1369979

[bib43] Schneider-Poetsch T, Ju J, Eyler DE, Dang Y, Bhat S, Merrick WC et al. Inhibition of eukaryotic translation elongation by cycloheximide and lactimidomycin. Nat Chem Biol 2010; 6: 209–217. 20118940 10.1038/nchembio.304 PMC2831214

[bib44] Wang Y, Shen J, Arenzana N, Tirasophon W, Kaufman R J, Prywes R. Activation of ATF6 and an ATF6 DNA Binding Site by the Endoplasmic Reticulum Stress Response. R. J Biol Chem 2000; 275: 27013–27020. 10856300 10.1074/jbc.M003322200

[bib45] Yoshida H, Haze K, Yanagi H, Yura T, Mori K. Identification of the *cis*-acting endoplasmic reticulum stress response element responsible for transcriptional induction of mammalian glucose-regulated proteins; involvement of basic leucine zipper transcription factors. J Biol Chem 1998; 273: 33741–33749. 9837962 10.1074/jbc.273.50.33741

[bib46] Anderson P, Kedersha N. Stressful initiations. J Cell Sci 2002; 115: 3227–3234. 12140254 10.1242/jcs.115.16.3227

[bib47] Shulman E, Belakhov V, Wei G, Kendall A, Meyron-Holtz EG, Ben-Shachar D et al. Designer aminoglycosides that selectively inhibit cytoplasmic rather than mitochondrial ribosomes show decreased ototoxicity: a strategy for the treatment of genetic diseases. J Biol Chem 2014; 289: 2318–2330. 24302717 10.1074/jbc.M113.533588 PMC3900975

[bib48] Xie J, Talaska AE, Schacht J. New developments in aminoglycoside therapy and ototoxicity. Hear Res 2011; 281: 28–37. 21640178 10.1016/j.heares.2011.05.008 PMC3169717

[bib49] Geisse S, Voedisch B. Transient expression technologies: past, present, and future. Methods Mol Biol 2012; 899: 203–219. 22735955 10.1007/978-1-61779-921-1_13

[bib50] Walter P, Ron D. The unfolded protein response: from stress pathway to homeostatic regulation. Science 2011; 334: 1081–1086. 22116877 10.1126/science.1209038

[bib51] Ozcan U, Cao Q, Yilmaz E, Lee AH, Iwakoshi NN, Ozdelen E et al. Endoplasmic reticulum stress links obesity, insulin action, and type 2 diabetes. Science 2004; 306: 457–461. 15486293 10.1126/science.1103160

[bib52] Francis SP, Katz J, Fanning KD, Harris KA, Nicholas BD, Lacy M, Pagana J, Agris PF, Shin JB. A novel role of cytosolic protein synthesis inhibition in aminoglycoside ototoxicity. J Neurosci 2013; 33: 3079–3093. 23407963 10.1523/JNEUROSCI.3430-12.2013 PMC3711767

[bib53] Taleb M, Brandon CS, Lee FS, Harris KC, Dillmann WH, Cunningham LL. Hsp70 inhibits aminoglycoside-induced hearing loss and cochlear hair cell death. Cell Stress Chaperones 2009; 14: 427–437. 19145477 10.1007/s12192-008-0097-2 PMC2728278

[bib54] Jiang H, Sha SH, Forge A, Schacht J. Caspase-independent pathways of hair cell death induced by kanamycin in vivo. Cell Death Differ 2006; 13: 20–30. 16021180 10.1038/sj.cdd.4401706 PMC1525047

[bib55] Fujinami Y, Mutai H, Mizutari K, Nakagawa S, Matsunaga T. A novel animal model of hearing loss caused by acute endoplasmic reticulum stress in the cochlea. J Pharmacol Sci 2012; 118: 363–372. 22362185 10.1254/jphs.11227fp

[bib56] Peyrou M, Hanna PE, Cribb AE. Cisplatin, gentamicin, and p-aminophenol induce markers of endoplasmic reticulum stress in the rat kidneys. Toxicol Sci 2007; 99: 346–353. 17567590 10.1093/toxsci/kfm152

[bib57] Kujawa SG, Liberman MC. Adding insult to injury: cochlear nerve degeneration after―temporary‖ noise-induced hearing loss. J Neurosci 2009; 29: 14077–14085. 19906956 10.1523/JNEUROSCI.2845-09.2009 PMC2812055

[bib58] Carrell RW, Lomas DA. Conformational disease. Lancet 1997; 350: 134–138. 9228977 10.1016/S0140-6736(97)02073-4

[bib59] Gidalevitz T, Kikis EA, Morimoto RI. A cellular perspective on conformational disease: the role of genetic background and proteostasis networks. Curr Opin Struct Biol 2010; 20: 23–32. 20053547 10.1016/j.sbi.2009.11.001 PMC3050498

[bib60] Matt T, Ng CL, Lang K, Sha SH, Akbergenov R, Shcherbakov D et al. Dissociation of antibacterial activity and aminoglycoside ototoxicity in the 4-monosubstituted 2-deoxystreptamine apramycin. Proc Natl Acad Sci USA 2012; 109: 10984–10989. 22699498 10.1073/pnas.1204073109 PMC3390888

[bib61] Salas-Marco J, Bedwell DM. Discrimination between defects in elongation fidelity and termination efficiency provides mechanistic insights into translational readthrough. J Mol Biol 2005; 348: 801–815. 15843014 10.1016/j.jmb.2005.03.025

[bib62] Kramer EB, Vallabhaneni H, Mayer LM, Farabaugh PJ. A comprehensive analysis of translational missense errors in the yeast Saccharomyces cerevisiae. RNA 2010; 16: 1797–1808. 20651030 10.1261/rna.2201210 PMC2924539

[bib63] Pfaffl MW. A new mathematical model for relative quantification in real-time RT-PCR. Nucleic Acids Res 2001; 29: e45. 11328886 10.1093/nar/29.9.e45 PMC55695

[bib64] Oishi N, Kendall A, Schacht J. Metformin protects against gentamicin-induced hair cell death in vitro but not ototoxicity in vivo. Neurosci Lett 2014; 583: 65–69. 25240593 10.1016/j.neulet.2014.09.028 PMC4253637

[bib65] Oishi N, Chen FQ, Zheng HW, Sha SH. Intra-tympanic delivery of short interfering RNA into the adult mouse cochlea. Hear Res 2013; 296: 36–41. 23183031 10.1016/j.heares.2012.10.011 PMC3557582

[bib66] Zhao LJ, Subramanian T, Zhou Y, Chinnadurai G. Acetylation by p300 regulates nuclear localization and function of the transcriptional corepressor CtBP2. J Biol Chem 2006; 281: 4183–4189. 16356938 10.1074/jbc.M509051200

[bib67] Tong M, Brugeaud A, Edge AS. Regenerated synapses between postnatal hair cells and auditory neurons. J Assoc Res Otolaryngol 2013; 14: 321–329. 23423560 10.1007/s10162-013-0374-3 PMC3642275

