# Supplementary material for: XBP1 mitigates aminoglycoside-induced endoplasmic reticulum stress and neuronal cell death
Source: Cell Death Dis. 2015 May 14;6(5):e1763–. doi: 10.1038/cddis.2015.108 (PMC4669688; doi:10.1038/cddis.2015.108)
Supplement: Supplementary Figures [file cddis2015108x1.pdf]

Supplementary Figures and Legends

Figure S1 Translation and viability assays

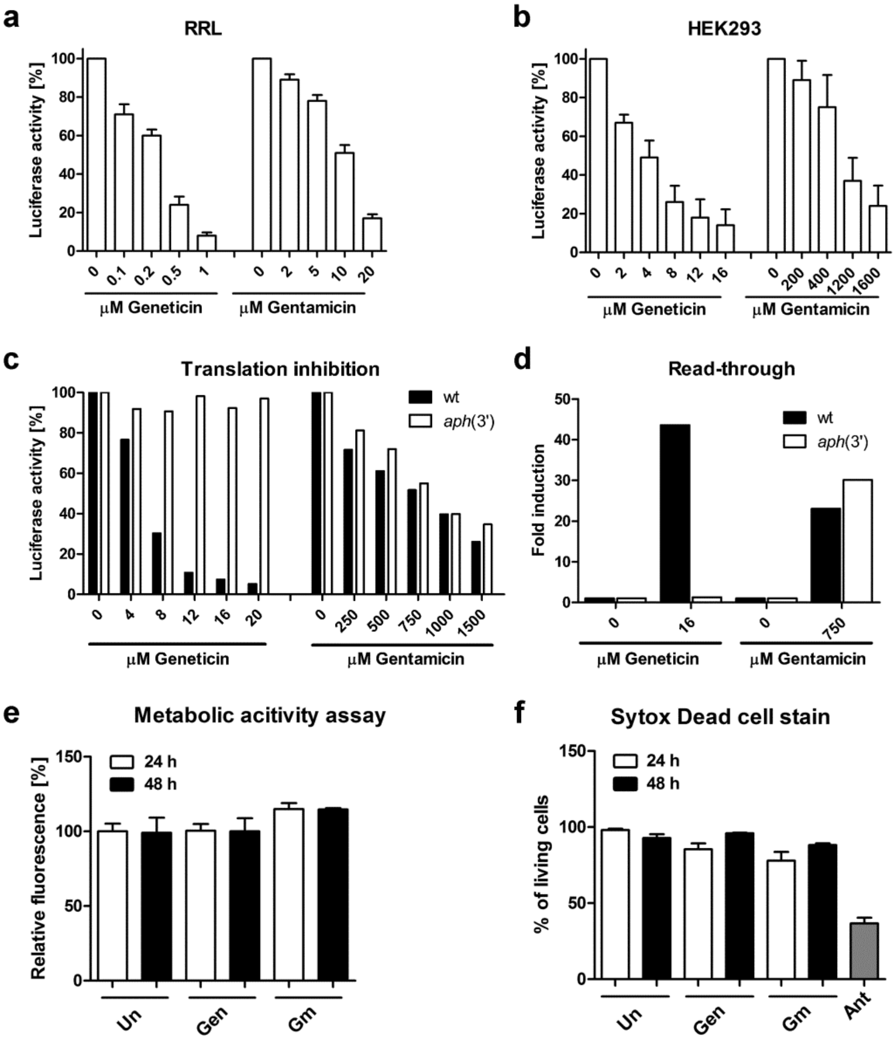

(**a–c**) Aminoglycoside-induced translation inhibition in (**a**) Rabbit reticulocyte lysate (RRL), (**b**) HEK wild-type cells, (**c**) HEK wild-type versus HEK *aph*(3') cells. Translation inhibition was measured by hRluc activity; hRluc signals of untreated samples are set as 100% luciferase activity. (**d**) Read-through, measured in HEK wild-type versus HEK *aph*(3') cells, indicated by the ratio hFluc/hRluc and given as fold induction. Untreated samples are set as 1. (**e**) Metabolic

activity assay. HEK wild-type cells were treated with geneticin (16 μM) or gentamicin (400 μM). The Alamar Blue fluorescence level of the untreated samples average was set as 100%. No statistical difference was observed between treated and untreated controls. (**f**) Sytox Dead cell stain. HEK wild-type cells were treated with geneticin (16 μM) or gentamicin (400 μM), stained with Sytox Red and analyzed by FACS. Antimycin (20 μg/mL for 8 h) was used as a positive control for cell death. The average fluorescence level of the untreated samples was set as 100% and percentage of living cells is presented. Treatment with geneticin and gentamicin slightly decreased cell viability by 5-15% ($p<0.05$) (**a, b, e, f**) Data are presented as means +SEM (*n*=3). Gen: Geneticin; Gm: Gentamicin; Ant: Antimycin; Un: Untreated.

Figure S2 Transcriptome analysis of geneticin-treated HEK wild-type cells

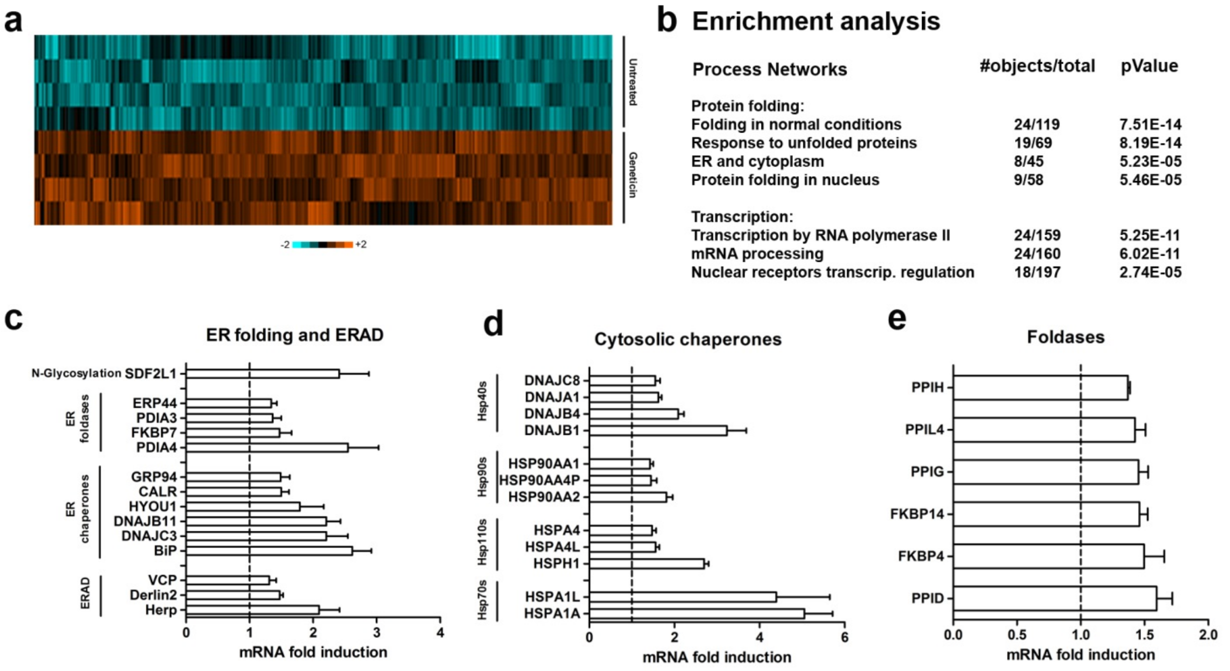

(**a**) Microarray analysis of geneticin-treated HEK cells compared to untreated control samples (*n*=4 in each) revealed 705 genes induced by geneticin (*Benjamini-Hochberg* corrected p-value

<0.05, FC >1.2). The figure represents a heat map of the genes. (**b**) Functional ontology enrichment analysis indicating the most significantly enriched networks (p-value <0.0001) of the 705 up-regulated genes. (**c, d, e**) mRNA fold induction of individual ER folding machinery and ERAD components (**c**), chaperones of the Hsp70, Hsp90, Hsp110, and Hsp40 protein families (**d**), and foldases (**e**) (*Benjamini-Hochberg* corrected p-value <0.05).

Methods. HEK cells were treated with 16 µM geneticin in F10 medium with 15 ug/mL saponin at 37 °C for 32 h. RNA was extracted from four independent samples for each condition (geneticin-treated, untreated). Biotinylated single-strand cDNA targets were prepared from 200 ng of total RNA, using the Ambion WT Expression Kit and the Affymetrix GeneChip WT Terminal Labeling Kit according to the manufacturer's protocols. Following fragmentation and end-labeling, 1.9 µg of cDNAs were hybridized for 16 h at 45 °C on GeneChip Human Gene 1.0 ST arrays (Affymetrix) interrogating 28,869 genes represented by approximately 27 probes spread across the full length of the gene. The chips were washed and stained in the GeneChip Fluidics Station 450 (Affymetrix) and scanned with the GeneChip® Scanner 3000 7G (Affymetrix) at a resolution of 0.7 µm. Raw data (.CEL intensity files) were extracted from the scanned images using the Affymetrix GeneChip Command Console, version 3.2. CEL files were further processed with Affymetrix Expression Console software version 1.1 to calculate probe set signal intensities using Robust Multi-array Average algorithms with default settings. Hierarchical clustering was performed using Cluster and TreeView software (http://www-microarrays.u-strasbg.fr). Functional ontology enrichment of process networks was analysed with the MetaCore software (GeneGo, Thomson Reuters). Significance of the difference in expression of each gene between treated and untreated samples was tested using the TREAT method, and the correction of Benjamini-Hochberg for multiple testing was applied in order to take into account the number of tests performed. Corrected p-values <0.05 were considered as significant.

Figure S3 Aminoglycoside-induced UPR in HeLa cells.

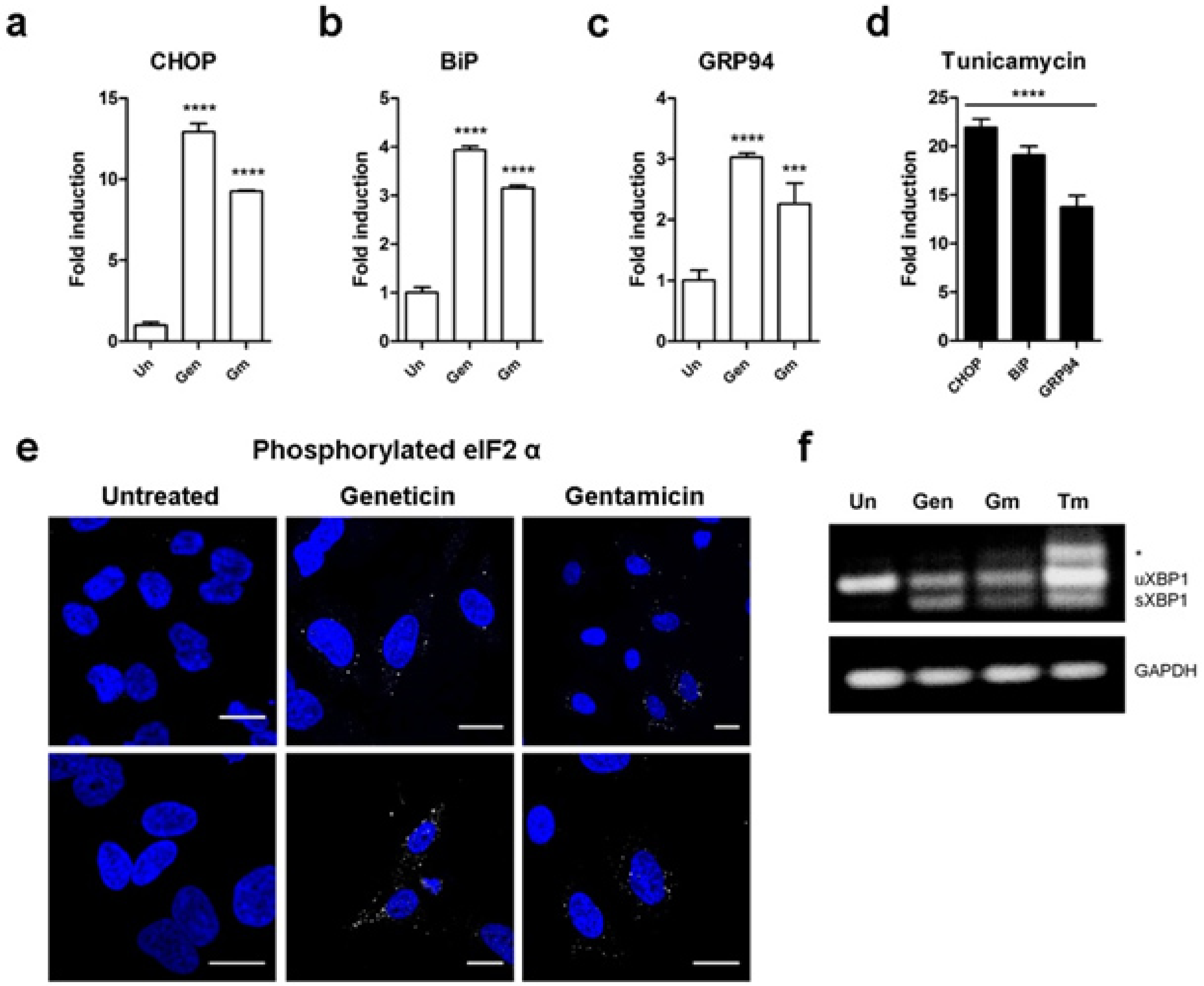

(**a–d**) qPCR analysis. HeLa wild-type cells were treated with geneticin (16 μM) or gentamicin (400 μM) and incubated for 24 h. Expression of mRNA of (**a**) CHOP, (**b**) BiP, and (**c**) GRP94 is shown. (**d**) Tunicamycin was used as a positive control. Experiments were run in triplicates and means +SD of fold induction relative to untreated samples are presented; ***$P<0.005$; ****$P<0.001$. (**e**) Phosphorylated eIF2α was detected by immunofluorescence. HeLa wild-type cells were treated with geneticin (16 μM) or gentamicin (400 μM) for 24 h. Scale bars: 20 μm. Two representative pictures are shown for each sample. (**f**) XBP1 splicing assay. HeLa wild-type cells were treated with geneticin (16 μM) or gentamicin (400 μM) for 24 h, or left untreated.

Products of XBP1 PCR were analyzed by gel electrophoresis; unspliced (uXBP1) and spliced (sXBP1) versions of XBP1 are indicated. Tunicamycin (2.5 µg/mL) was a positive control to induce ER stress; GAPDH was a loading control. The asterisk indicates the position of a hybrid amplicon[15]. Gen: Geneticin; Gm: Gentamicin; Tm: Tunicamycin; UN: Untreated.

Figure S4 Effect of tunicamycin on cochlear explants

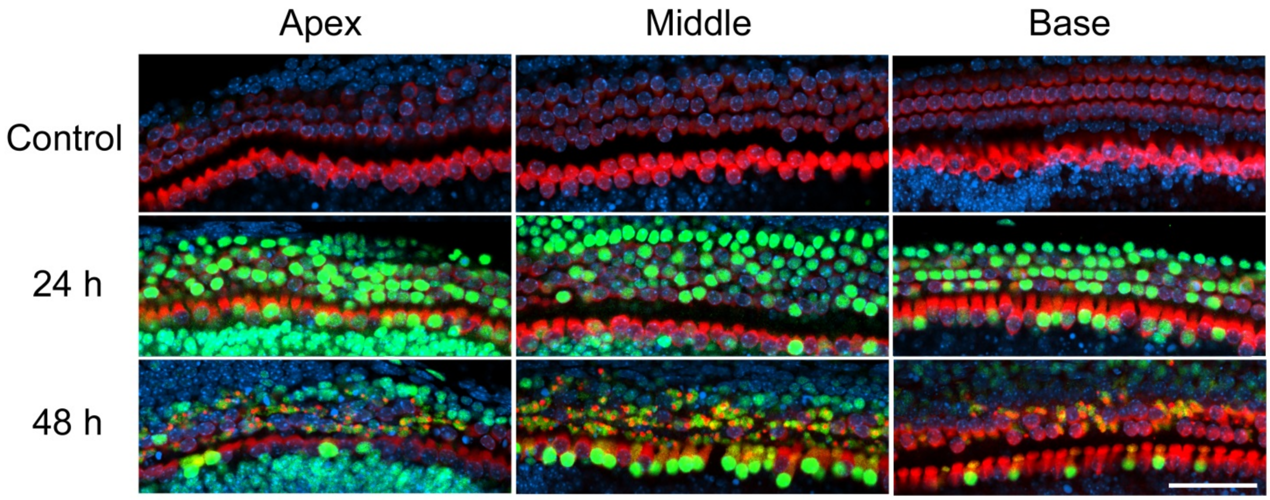

Control: The specific ER stress-associated pro-apoptotic factor, CHOP (green) is absent from untreated organ of Corti explants of CBA/J mice (P2–3). **24 h:** After a 24-h incubation, tunicamycin (0.07 µg/mL) induced CHOP in the nuclei of most hair cells from base to apex of the organ of Corti. **48 h:** Loss of staining consistent with beginning hair cell death. Green: CHOP (GADD 153 antibody), red: anti-myo7a stain for hair cells, blue: Hoechst 33342 staining for nuclei. The figure represents three different explants at each time point. Scale bar: 50 µm.

Figure S5 Effect of gentamicin on hair cells *in vitro* and *in vivo*

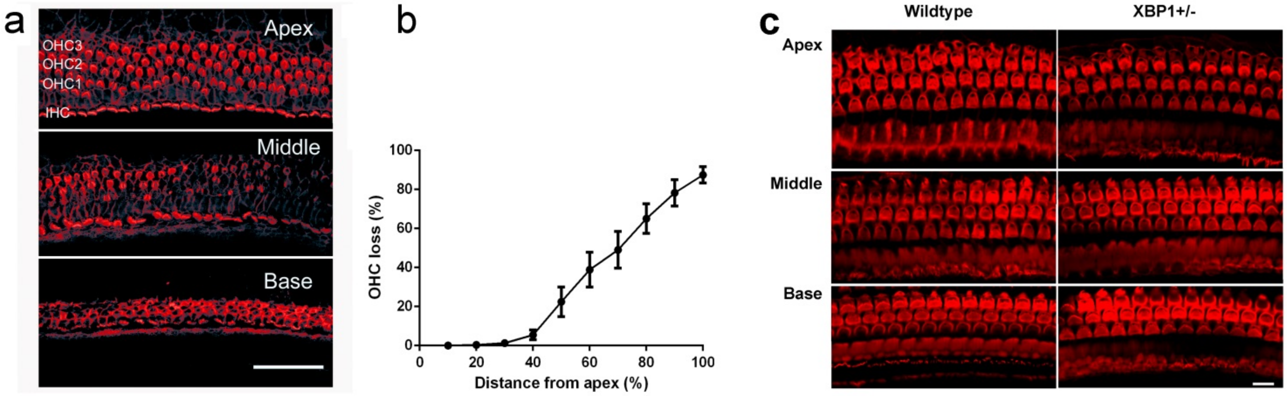

(a, b): Effect of gentamicin on hair cells in cochlear explants. (**a**) Loss of OHCs due to gentamicin treatment (3.5 μM for 72 h) showed the typical base-to-apex gradient with most destruction in the base. The figure represents six different explants from CBA/J mice. (**b**) Complete quantification of hair cell loss from apex to base of the explant. Data are mean $\underline{+}$SD. Red: rhodamine phalloidin to outline hair cell structure. Scale bar: 50 μm.

(c): Effect of intratympanic application of gentamicin on cochlear hair cells. Surface preparations from adult wild-type and XBP1$^{+/-}$ mice treated with intratympanically applied gentamicin were examined from base to apex. Actin staining (red) showed the presence of OHC, cuticular plates and stereocilia in all parts of the cochlea, except for minor scattered loss of cells at the base. The images are representative samples of five wild-type and five XBP1$^{+/-}$ mice treated with gentamicin. Scale bar: 10 μm.

Figure S6 Distortion product otoacoustic emissions (DPOAE) remain unaltered by intratympanic gentamicin treatment

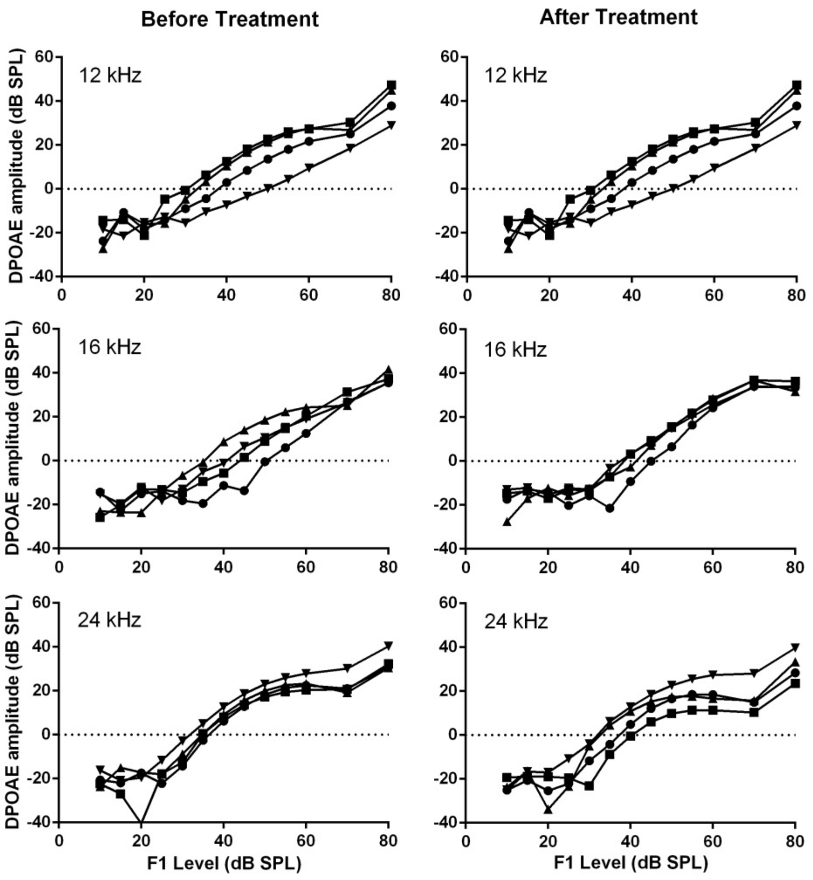

Animals were anesthetized with ketamine 65 mg/kg, xylazine 3.5 mg/kg, and acepromazine 2 mg/kg and body temperature was maintained. The primary tones, F1 and F2, were set at a F2/F1 ratio of 1.2. The intensity of F1 (L1) was varied in 5- or 10-dB steps (with the intensity of F1 ranging from 10–80 dB SPL), and the intensity of F2 (L2) was maintained 10 dB lower than L1. DPOAE were measured at 2F1 - F2. Tones were presented via two EC1 drivers (TDT) connected through an electret condenser microphone (Knowles Acoustics, type FG-23329-P07). TDT System III hardware and SigGen/BioSig software were used to present the stimuli and record responses. DPOAE responses in four adult XBP1$^{+/-}$ mice were measured before and after gentamicin treatment. Lines for the four animals are distinguished by different symbols.
