# Supplementary material for: XBP1 mitigates aminoglycoside-induced endoplasmic reticulum stress and neuronal cell death
Source: Cell Death Dis. 2015 May 14;6(5):e1763–. doi: 10.1038/cddis.2015.108 (PMC4669688; doi:10.1038/cddis.2015.108)
Supplement: Supplementary Table S1 [file cddis2015108x2.pdf]

**Supplementary Table 1.**

List of analyzed UPR and ER folding machinery genes and human chaperones and foldases.
* Genes considered significantly regulated (p-value ≤ BH correction)

| Gene symbol | Other symbols | Fold change | P-value | BH correction |
|---|---|---|---|---|
| **UPR**[1-3]<br>UPR sensors | | | | |
| ERN1 | IRE1 | 0.88 | 0.193 | 0.013 |
| EIF2AK3 | PERK | 1.26 | 0.033 | 0.006 |
| ATF6 | | 1.03 | 0.778 | 0.034 |
| ATF6B | | 0.68 | 0.001* | 0.001 |
| UPR Transcription factors | | | | |
| ATF3 | | 0.99 | 0.898 | 0.041 |
| ATF4 | | 0.82 | 0.013 | 0.004 |
| ATF6 | | 1.03 | 0.778 | 0.034 |
| ATF6B | | 0.68 | 0.001* | 0.001 |
| XBP1 | | 1.30 | 0.012 | 0.004 |
| DDIT3 | CHOP | 1.51 | 0.020 | 0.005 |
| **ERAD**[4]<br>Processing and targeting | | | | |
| EDEM1 | | 0.97 | 0.913 | 0.042 |
| EDEM2 | | 1.04 | 0.806 | 0.036 |
| EDEM3 | | 1.12 | 0.182 | 0.012 |
| PDIA2 | PDI, PDIP, PDIR | 0.91 | 0.458 | 0.022 |
| HSPA5 | BiP, GRP78 | 2.62 | <0.001* | <0.001 |
| HSP90B1 | GRP94 | 1.49 | <0.001* | <0.001 |
| DNAJB9 | ERdj4, MDG1 | 1.78 | 0.003 | 0.002 |
| DNAJC10 | ERdj5, JPD1 | 0.82 | 0.015 | 0.004 |
| FOXRED2 | ERFAD | 0.74 | <0.001* | <0.001 |
| PPIB | Cyclophilin B, CYPB | 1.00 | 0.987 | 0.049 |
| OS9 | ERLEC2 | 0.96 | 0.724 | 0.032 |
| ERLEC1 | XTP3-B | 1.21 | 0.037 | 0.006 |
| SEL1L | | 1.25 | 0.061 | 0.007 |
| Possible retrotranslocation channel | | | | |
| SEC61A1 | SEC61 | 1.03 | 0.845 | 0.038 |
| SEC61A2 | | 1.13 | 0.300 | 0.017 |
| SEC61B | | 1.14 | 0.141 | 0.011 |
| SEC61G | | 1.57 | 0.001* | 0.001 |
| DERL1 | Derlin 1 | 1.08 | 0.458 | 0.022 |
| DERL2 | Derlin 2 | 1.48 | <0.001* | <0.001 |
| DERL3 | Derlin 3 | 1.43 | 0.008 | 0.003 |

| | | | | |
|---|---|---|---|---|
| Other possible component or regulator | | | | |
| HERPUD1 | HERP, Mif1, SUP | 2.10 | <0.001* | <0.001 |
| VIMP | SELS | 1.43 | 0.004 | 0.002 |
| BCAP31 | BAP31 | 1.09 | 0.442 | 0.021 |
| JKAMP | HSPC213, JAMP | 1.20 | 0.011 | 0.004 |
| DNAJB12 | DJ10 | 0.99 | 0.961 | 0.046 |
| HM13 | SPP | 0.99 | 0.949 | 0.045 |
| SSR1 | TRAP alpha | 0.87 | 0.114 | 0.010 |
| SSR2 | TRAP beta | 1.00 | 0.984 | 0.049 |
| SSR3 | TRAP gamma | 0.97 | 0.828 | 0.037 |
| SSR4 | TRAP delta | 0.88 | 0.176 | 0.012 |
| TICAM2 | TRAM | 1.17 | 0.082 | 0.008 |
| AUP1 | | 1.22 | 0.002 | 0.002 |
| SVIP | | 1.15 | 0.212 | 0.013 |
| E2 ubiquitin-conjugating enzyme | | | | |
| UBE2K | UBC1, HIP2 | 1.20 | 0.008 | 0.003 |
| UBE2D1 | UBCH5 | 1.42 | <0.001* | <0.001 |
| UBE2J1 | UBC6 | 0.96 | 0.725 | 0.032 |
| UBE2J2 | | 1.14 | 0.154 | 0.011 |
| UBE2G1 | UBC7 | 1.04 | 0.788 | 0.035 |
| UBE2G2 | | 1.17 | 0.090 | 0.009 |
| UBE2N | UBC13 | 1.11 | 0.233 | 0.014 |
| E3 ubiquitin-ligase | | | | |
| NEDD4L | NEDD4-2 | 1.15 | 0.135 | 0.011 |
| PARK2 | PDJ | 0.67 | 0.001* | 0.001 |
| RNF5 | RMA1 | 1.34 | 0.001* | 0.001 |
| AMFR | RNF45, GP78 | 0.95 | 0.638 | 0.028 |
| SYVN1 | HRD1, DER3 | 1.28 | 0.007 | 0.003 |
| MARCH6 | TEB4, DOA10 | 0.96 | 0.772 | 0.034 |
| RNF139 | HRCA1, TRC8 | 1.27 | 0.006 | 0.003 |
| TRIM13 | CAR, RNF77 | 1.01 | 0.971 | 0.047 |
| RNF103 | KF1 | 1.09 | 0.343 | 0.018 |
| RNF19A | RNF19 | 1.12 | 0.212 | 0.013 |
| RNF121 | | 1.36 | 0.003 | 0.002 |
| STUB1 | CHIP | 0.95 | 0.679 | 0.030 |
| SKP1-CUL1-F-box (SCF) E3 | | | | |
| SKP1 | OCP | 1.36 | 0.006 | 0.003 |
| CUL1 | cullin-1 | 1.23 | 0.004 | 0.002 |
| FBXO2 | FBG1 | 0.88 | 0.160 | 0.011 |
| FBXO6 | FBG2 | 0.91 | 0.247 | 0.015 |
| RBX1 | RNF75, ROC1 | 1.09 | 0.357 | 0.018 |

| | | | | |
|---|---|---|---|---|
| E4 ubiquitin-conjugating enzyme | | | | |
| UBE4B | UFD2 | 0.90 | 0.240 | 0.014 |
| Substrate extraction and recruiting | | | | |
| VCP | p97, ALS14 | 1.31 | 0.001* | 0.002 |
| UFD1L | UFD1 | 1.30 | 0.003 | 0.002 |
| NPLOC4 | NPL4 | 1.13 | 0.187 | 0.012 |
| UBXD family protein | | | | |
| UBXN6 | UBXD1 | 1.02 | 0.905 | 0.042 |
| UBXN4 | UBXD2 | 1.12 | 0.226 | 0.014 |
| UBXN7 | UBXD7 | 1.31 | 0.010 | 0.003 |
| FAF2 | UBXD8 | 1.47 | <0.001* | 0.001 |
| UBXN1 | UBXD10 | 1.02 | 0.844 | 0.038 |
| Deglycosylating enzyme | | | | |
| NGLY1 | PNGase | 1.11 | 0.270 | 0.015 |
| DUB (deubiquitination) | | | | |
| VCPIP1 | DUBA3, VCIP135 | 1.21 | 0.011 | 0.004 |
| YOD1 | DUBA8, YOD1 | 1.57 | <0.001* | <0.001 |
| ATXN3 | Ataxin-3 | 1.12 | 0.259 | 0.015 |
| USP19 | | 1.19 | 0.071 | 0.008 |
| Shuttle protein | | | | |
| UBQLN1 | Ubiquilin1 | 1.18 | 0.017 | 0.004 |
| RAD23A | HR23A | 0.78 | 0.004 | 0.003 |
| RAD23B | HR23B | 0.91 | 0.382 | 0.019 |
| Ubiquitin receptor | | | | |
| PSMD4 | Rpn10 | 0.86 | 0.159 | 0.011 |
| PSMC3 | Rpt5 | 0.95 | 0.686 | 0.030 |
| ADRM1 | Rpn13 | 1.13 | 0.247 | 0.015 |
| **ER Chaperones**[2, 4, 5] | | | | |
| DNAJC1 | ERdj1, MTJ1 | 0.97 | 0.834 | 0.037 |
| SEC63 | ERdj2, DNAJC23 | 0.91 | 0.343 | 0.018 |
| DNAJB11 | ERdj3, HEDJ, ERj3 | 2.21 | <0.001* | <0.001 |
| DNAJB9 | ERdj4, MDG1 | 1.78 | 0.003 | 0.002 |
| DNAJC10 | ERdj5, JPD1 | 0.82 | 0.015 | 0.004 |
| TOR1A | Torsin A | 1.05 | 0.636 | 0.028 |
| SIL1 | BAP, ULG5 | 1.02 | 0.886 | 0.040 |
| HYOU1 | GRP170 | 1.79 | <0.001* | 0.001 |
| HSP90B1 | GRP94 | 1.49 | <0.001* | <0.001 |
| HSPA5 | BiP | 2.62 | <0.001* | <0.001 |

| | | | | |
|---|---|---|---|---|
| CALR | Calreticulin | 1.50 | <0.001* | 0.001 |
| CANX | Calnexin | 1.10 | 0.310 | 0.017 |
| SERPINH1 | HSP47 | 1.24 | 0.024 | 0.005 |
| LRPAP1 | RAP | 1.07 | 0.558 | 0.025 |
| LEPRE1 | P3H1 | 0.95 | 0.641 | 0.028 |
| P4HB | P4H, ERP59, PDIA1 | 0.95 | 0.668 | 0.029 |
| DNAJC3 | ERdj6 | 2.21 | <0.001* | <0.001 |
| **ER foldases**[2, 5-7] | | | | |
| PDIA3 | ERP57, ERP61, ERP60 | 1.36 | 0.001* | 0.002 |
| PDIA4 | ERP70, ERP72 | 2.55 | <0.001* | <0.001 |
| DNAJC10 | ERdj5 | 0.82 | 0.015 | 0.004 |
| PDIA5 | PDIR | 0.97 | 0.832 | 0.037 |
| MUTED | ERP46, PDIA15 | 0.98 | 0.903 | 0.042 |
| PDIA2 | PDI, PDIp, PDIr | 0.91 | 0.458 | 0.022 |
| PDILT | PDIA7 | 0.93 | 0.507 | 0.024 |
| ERP44 | PDIA10, TXNDC4 | 1.34 | 0.001* | 0.001 |
| TXNDC12 | ERP18, PDIA16, ERP19 | 1.17 | 0.035 | 0.006 |
| TMX1 | TMX, PDIA11 | 1.06 | 0.589 | 0.026 |
| TMX2 | PDIA12 | 1.26 | 0.021 | 0.005 |
| TMX3 | PDIA13 | 0.96 | 0.755 | 0.033 |
| TMX4 | PDIA14 | 0.90 | 0.297 | 0.016 |
| PDIA6 | P5, ERP5, TXNDC7 | 1.42 | 0.002 | 0.002 |
| ERO1LB | ERO1B | 1.60 | 0.003 | 0.002 |
| ERO1L | ERO1A | 0.79 | 0.004 | 0.002 |
| P4HB | PDIA1, ERP59 | 0.95 | 0.668 | 0.029 |
| ERP29 | PDIA9, ERP28, ERP31 | 0.67 | <0.001* | 0.001 |
| PPIB | Cyclophilin B, CYPB | 1.00 | 0.987 | 0.049 |
| FKBP2 | FKBP13 | 1.05 | 0.717 | 0.032 |
| FKBP7 | FKBP23 | 1.47 | <0.001* | 0.001 |
| FKBP10 | FKBP65 | 0.74 | 0.002 | 0.002 |
| FKBP11 | FKBP19 | 1.14 | 0.370 | 0.019 |
| **N-linked Glycosylation**[2, 7] | | | | |
| UGGT1 | UGT1 | 1.00 | 0.973 | 0.047 |
| UGGT2 | UGT2 | 1.09 | 0.478 | 0.023 |
| SDF2 | | 1.50 | 0.003 | 0.002 |
| SDF2L1 | | 2.41 | <0.001* | <0.001 |
| MOGS | alpha glucosidase I | 1.11 | 0.366 | 0.019 |
| GANAB | alpha glucosidase II | 0.80 | 0.006 | 0.003 |
| MAN1A1 | alpha mannosidase I | 0.99 | 0.953 | 0.046 |
| MAN2A1 | alpha mannosidase II | 0.81 | 0.023 | 0.005 |
| ALG12 | | 1.29 | 0.008 | 0.003 |

| | | | | |
|---|---|---|---|---|
| ALG5 | | 1.30 | 0.006 | 0.003 |
| PIGA | GPI3 | 1.35 | 0.012 | 0.004 |
| PIGB | | 0.87 | 0.169 | 0.012 |
| RPN1 | OST1 | 1.01 | 0.942 | 0.045 |
| STT3A | STT3, ITM1 | 0.90 | 0.226 | 0.014 |
| DDOST | WBP1 | 0.95 | 0.653 | 0.029 |
| **Human Chaperones**[8]<br>HSPA (Hsp70 chaperones) | | | | |
| HSPA1A | | 5.05 | <0.001* | <0.001 |
| HSPA1L | | 4.40 | <0.001* | <0.001 |
| HSPA2 | | 1.37 | 0.005 | 0.003 |
| HSPA5 | BiP, GRP78 | 2.62 | <0.001* | <0.001 |
| HSPA6 | | 1.65 | 0.004 | 0.002 |
| HSPA7 | | 1.65 | 0.004 | 0.002 |
| HSPA8 | | 1.17 | 0.021 | 0.005 |
| HSPA9 | | 1.00 | 0.977 | 0.048 |
| HSPA12A | | 0.92 | 0.381 | 0.019 |
| HSPA12B | | 0.96 | 0.680 | 0.030 |
| HSPA13 | | 1.05 | 0.715 | 0.031 |
| HSPA14 | | 1.26 | 0.007 | 0.003 |
| HSPH (Hsp110 chaperones) | | | | |
| HYOU1 | GRP170 | 1.79 | <0.001* | 0.001 |
| HSPH1 | | 2.70 | <0.001* | <0.001 |
| HSPA4 | | 1.48 | <0.001* | <0.001 |
| HSPA4L | | 1.56 | <0.001* | <0.001 |
| HSPC (Hsp90 chaperones) | | | | |
| HSP90AA1 | | 1.43 | <0.001* | <0.001 |
| HSP90AA2 | | 1.81 | <0.001* | <0.001 |
| HSP90AB1 | | 1.06 | 0.651 | 0.029 |
| HSP90B1 | GRP94 | 1.49 | <0.001* | <0.001 |
| TRAP1 | | 0.82 | 0.016 | 0.004 |
| DnaJA (Hsp40 co-chaperones) | | | | |
| DNAJA1 | | 1.62 | <0.001* | <0.001 |
| DNAJA2 | | 1.14 | 0.121 | 0.010 |
| DNAJA3 | | 1.12 | 0.169 | 0.012 |
| DNAJA4 | | 0.99 | 0.938 | 0.044 |
| DnaJB (Hsp40 co-chaperones) | | | | |
| DNAJB1 | | 3.23 | <0.001* | <0.001 |
| DNAJB2 | | 0.90 | 0.376 | 0.019 |

| | | | | |
|---|---|---|---|---|
| DNAJB3 | | 0.93 | 0.517 | 0.024 |
| DNAJB4 | | 2.09 | <0.001* | <0.001 |
| DNAJB5 | | 1.10 | 0.321 | 0.017 |
| DNAJB6 | | 1.22 | 0.018 | 0.004 |
| DNAJB7 | | 0.99 | 0.921 | 0.043 |
| DNAJB8 | | 0.90 | 0.217 | 0.014 |
| DNAJB9 | | 1.78 | 0.003 | 0.002 |
| DNAJB11 | | 2.21 | <0.001* | <0.001 |
| DNAJB12 | | 0.99 | 0.961 | 0.046 |
| DNAJB13 | | 1.00 | 0.963 | 0.047 |
| DNAJB14 | | 1.04 | 0.721 | 0.032 |
| DnaJC (Hsp40 co-chaperones) | | | | |
| DNAJC1 | | 0.97 | 0.834 | 0.037 |
| DNAJC2 | MPP11 | 1.19 | 0.021 | 0.005 |
| DNAJC3 | ERdj6 | 2.21 | <0.001* | <0.001 |
| DNAJC4 | | 0.82 | 0.020 | 0.005 |
| DNAJC5 | | 1.05 | 0.693 | 0.030 |
| DNAJC5B | | 0.99 | 0.946 | 0.045 |
| DNAJC5G | | 1.00 | 0.984 | 0.049 |
| DNAJC6 | | 1.31 | 0.006 | 0.003 |
| DNAJC7 | | 1.10 | 0.223 | 0.014 |
| DNAJC8 | | 1.55 | 0.001* | 0.001 |
| DNAJC9 | | 1.22 | 0.028 | 0.005 |
| DNAJC10 | ERdj5 | 0.82 | 0.015 | 0.004 |
| DNAJC11 | | 1.38 | 0.008 | 0.003 |
| DNAJC12 | | 1.11 | 0.372 | 0.019 |
| DNAJC13 | | 0.96 | 0.781 | 0.035 |
| DNAJC14 | | 1.01 | 0.924 | 0.043 |
| DNAJC15 | | 0.98 | 0.874 | 0.040 |
| DNAJC16 | | 0.80 | 0.015 | 0.004 |
| DNAJC17 | | #N/A | #N/A | #N/A |
| DNAJC18 | | 0.75 | 0.001* | 0.001 |
| DNAJC19 | TIMM14 | 0.98 | 0.897 | 0.041 |
| DNAJC21 | | 1.03 | 0.769 | 0.034 |
| DNAJC22 | | 0.98 | 0.868 | 0.039 |
| DNAJC24 | DPH4 | 1.15 | 0.112 | 0.010 |
| DNAJC25 | | 1.19 | 0.023 | 0.005 |
| DNAJC27 | | 1.12 | 0.349 | 0.018 |
| DNAJC28 | | 0.96 | 0.703 | 0.031 |
| DNAJC30 | | 0.99 | 0.972 | 0.047 |
| HSCB | | 1.04 | 0.776 | 0.034 |
| SEC63 | ERdj2 | 0.91 | 0.343 | 0.018 |
| GAK | DNAJC26 | 1.06 | 0.638 | 0.028 |
| SACS | DNAJC29 | 1.10 | 0.328 | 0.017 |

| | | | | |
|---|---|---|---|---|
| HspB (small heat shock proteins) | | | | |
| HSPB1 | HSP25 | 1.90 | 0.001* | 0.001 |
| HSPB2 | HSP27 | 0.93 | 0.532 | 0.024 |
| HSPB3 | HSPL27 | 1.07 | 0.611 | 0.027 |
| HSPB6 | HSP20 | #N/A | #N/A | #N/A |
| HSPB7 | | 0.92 | 0.466 | 0.022 |
| HSPB8 | | 1.42 | 0.121 | 0.010 |
| HSPB9 | | 0.91 | 0.356 | 0.018 |
| HSPB11 | | 1.11 | 0.290 | 0.016 |
| HSPBAP1 | | 1.18 | 0.049 | 0.007 |
| CRYAA | | 0.88 | 0.224 | 0.014 |
| CRYAB | | 1.03 | 0.811 | 0.036 |
| Chaperonin (Hsp10 and 60) | | | | |
| HSPD1 | HSP60, GROEL | 1.15 | 0.036 | 0.006 |
| HSPD1P1 | | 1.36 | 0.010 | 0.003 |
| HSPE1 | HSP10, GROES | 1.58 | <0.001* | <0.001 |
| MKKS | | 0.99 | 0.940 | 0.045 |
| BBS10 | | 1.30 | 0.012 | 0.004 |
| BBS12 | | 1.15 | 0.260 | 0.015 |
| Chaperone regulator | | | | |
| STIP1 | | 1.72 | <0.001* | <0.001 |
| CLIPs (ribosome-associated chaperones) | | | | |
| TCPA1 | | 1.19 | 0.005 | 0.003 |
| CCT2 | | 1.07 | 0.525 | 0.024 |
| CCT3 | | 1.13 | 0.133 | 0.010 |
| CCT4 | | 1.10 | 0.264 | 0.015 |
| CCT5 | | 1.14 | 0.047 | 0.007 |
| CCT6A | | 1.26 | <0.001* | 0.001 |
| CCT6B | | 0.97 | 0.855 | 0.039 |
| CCT7 | | 1.13 | 0.089 | 0.009 |
| CCT8 | | 1.10 | 0.292 | 0.016 |
| PFDN1 | | 1.22 | 0.007 | 0.003 |
| PFDN2 | | 1.15 | 0.088 | 0.009 |
| VBP1 | | 1.29 | 0.009 | 0.003 |
| PFDN4 | | 1.21 | 0.048 | 0.007 |
| PFDN5 | | 0.99 | 0.963 | 0.047 |
| PFDN6 | | 1.48 | <0.001* | 0.001 |
| DNAJC2 | | 1.19 | 0.021 | 0.005 |
| HSPA14 | | 1.26 | 0.007 | 0.003 |
| BTF3 | | 1.01 | 0.956 | 0.046 |
| NACA | | 1.01 | 0.955 | 0.046 |

| | | | | |
|---|---|---|---|---|
| **Peptidylprolyl cis-trans Isomerases (PPI)**[9, 10] | | | | |
| FKBP1A | | 1.04 | 0.056 | 0.007 |
| FKBP1B | | 1.14 | 0.197 | 0.013 |
| FKBP2 | | 1.05 | 0.717 | 0.032 |
| FKBP3 | | 1.04 | 0.723 | 0.032 |
| FKBP4 | | 1.50 | <0.001* | 0.001 |
| FKBP5 | | 1.27 | 0.003 | 0.002 |
| FKBP6 | | 0.88 | 0.206 | 0.013 |
| FKBP7 | | 1.47 | <0.001* | 0.001 |
| FKBP8 | | 0.97 | 0.817 | 0.036 |
| FKBP9 | | 0.83 | 0.060 | 0.007 |
| FKBP9L | | 0.81 | 0.023 | 0.005 |
| FKBP10 | | 0.74 | 0.002 | 0.002 |
| FKBP11 | | 1.14 | 0.370 | 0.019 |
| FKBP14 | | 1.46 | 0.001* | 0.001 |
| FKBPL | | 1.02 | 0.843 | 0.038 |
| PIN1 | | 1.15 | 0.073 | 0.008 |
| PIN1P1 | | #N/A | #N/A | #N/A |
| PIN4 | | 1.18 | 0.154 | 0.011 |
| PPIA | | 1.08 | 0.418 | 0.020 |
| PPIAL4A | | 1.02 | 0.927 | 0.044 |
| PPIAL4B | | 1.00 | 0.927 | 0.044 |
| PPIAL4C | | 1.00 | 0.927 | 0.044 |
| PPIAL4D | | #N/A | #N/A | #N/A |
| PPIAL4E | | 1.00 | 0.927 | 0.044 |
| PPIAL4F | | 1.00 | 0.927 | 0.044 |
| PPIAL4G | | 0.97 | 0.908 | 0.042 |
| PPIB | Cyclophilin B, CYPB | 1.00 | 0.987 | 0.049 |
| PPIC | | 0.92 | 0.432 | 0.021 |
| PPID | | 1.59 | <0.001* | 0.001 |
| PPIE | | 1.19 | 0.077 | 0.008 |
| PPIEL | | 1.04 | 0.949 | 0.045 |
| PPIF | | 1.00 | 0.992 | 0.049 |
| PPIG | | 1.45 | <0.001* | 0.001 |
| PPIH | | 1.37 | <0.001* | <0.001 |
| PPIL1 | | 1.17 | 0.045 | 0.007 |
| PPIL2 | | 0.82 | 0.010 | 0.003 |
| PPIL3 | | 0.91 | 0.304 | 0.017 |
| PPIL4 | | 1.42 | 0.001* | 0.001 |
| PPIL6 | | 0.85 | 0.095 | 0.009 |
| PPWD1 | | 1.10 | 0.325 | 0.017 |

| Protein Disulfide Isomerases (PDI)[11] | | | | |
|---|---|---|---|---|
| PDIA2 | | 0.91 | 0.458 | 0.022 |
| PDIA3 | | 1.36 | 0.001* | 0.002 |
| PDIA4 | | 2.55 | <0.001* | <0.001 |
| PDIA5 | | 0.97 | 0.832 | 0.037 |
| PDIA6 | | 1.42 | 0.002 | 0.002 |
| PDILT | | 0.93 | 0.507 | 0.024 |
| PDIK1L | | 0.93 | 0.502 | 0.023 |
| P4HB | | 0.95 | 0.668 | 0.029 |
| ERP27 | | 0.97 | 0.880 | 0.040 |
| ERP29 | | 0.67 | <0.001* | 0.001 |
| ERP44 | | 1.34 | 0.001* | 0.001 |
| TMX1 | | 1.06 | 0.589 | 0.026 |
| TMX2 | | 1.26 | 0.021 | 0.005 |
| TMX3 | | 0.96 | 0.755 | 0.033 |
| TMX4 | | 0.90 | 0.297 | 0.016 |
| TXNDC5 | | 1.21 | 0.274 | 0.016 |
| TXNDC12 | | 1.17 | 0.035 | 0.006 |
| AGR2 | | 1.07 | 0.528 | 0.024 |
| AGR3 | | 0.97 | 0.898 | 0.041 |
| DNAJC10 | | 0.82 | 0.015 | 0.004 |
| CASQ1 | | 0.98 | 0.930 | 0.044 |
| CASQ2 | | 0.92 | 0.397 | 0.020 |

## References


1. Hetz, C. The unfolded protein response: controlling cell fate decisions under ER stress and beyond. *Nat Rev Mol Cell Biol* **13**, 89-102 (2012).
2. Lecca, M.R., Wagner, U., Patrignani, A., Berger, E.G. & Hennet, T. Genome-wide analysis of the unfolded protein response in fibroblasts from congenital disorders of glycosylation type-I patients. *FASEB J* **19**, 240-242 (2005).
3. Sharma, R., Jiang, H., Zhong, L., Tseng, J. & Gow, A. Minimal role for activating transcription factor 3 in the oligodendrocyte unfolded protein response in vivo. *J Neurochem* **102**, 1703-1712 (2007).
4. Araki, K. & Nagata, K. Protein folding and quality control in the ER. *Cold Spring Harb Perspect Biol* **3**, a007526 (2011).
5. Hebert, D.N. & Molinari, M. In and out of the ER: protein folding, quality control, degradation, and related human diseases. *Physiol Rev* **87**, 1377-1408 (2007).
6. Bernasconi, R. & Molinari, M. ERAD and ERAD tuning: disposal of cargo and of ERAD regulators from the mammalian ER. *Curr Opin Cell Biol* **23**, 176-183 (2011).
7. Schroder, M. & Kaufman, R.J. The mammalian unfolded protein response. *Annu Rev Biochem* **74**, 739-789 (2005).
8. Kampinga, H.H. *et al.* Guidelines for the nomenclature of the human heat shock proteins. *Cell Stress Chaperones* **14**, 105-111 (2009).
9. Gerard, M., Deleersnijder, A., Demeulemeester, J., Debyser, Z. & Baekelandt, V. Unraveling the role of peptidyl-prolyl isomerases in neurodegeneration. *Mol Neurobiol* **44**, 13-27 (2011).
10. Benham, A.M. The protein disulfide isomerase family: key players in health and disease. *Antioxid Redox Signal* **16**, 781-789 (2012).
